# Evolutionary Triplet Models of Structured RNA

**DOI:** 10.1371/journal.pcbi.1000483

**Published:** 2009-08-28

**Authors:** Robert K. Bradley, Ian Holmes

**Affiliations:** 1 Biophysics Graduate Group, University of California, Berkeley, California, United States of America; 2 Department of Bioengineering, University of California, Berkeley, California, United States of America; Washington University School of Medicine, United States of America

## Abstract

The reconstruction and synthesis of ancestral RNAs is a feasible goal for paleogenetics. This will require new bioinformatics methods, including a robust statistical framework for reconstructing histories of substitutions, indels and structural changes. We describe a “transducer composition” algorithm for extending pairwise probabilistic models of RNA structural evolution to models of multiple sequences related by a phylogenetic tree. This algorithm draws on formal models of computational linguistics as well as the 1985 protosequence algorithm of David Sankoff. The output of the composition algorithm is a multiple-sequence stochastic context-free grammar. We describe dynamic programming algorithms, which are robust to null cycles and empty bifurcations, for parsing this grammar. Example applications include structural alignment of non-coding RNAs, propagation of structural information from an experimentally-characterized sequence to its homologs, and inference of the ancestral structure of a set of diverged RNAs. We implemented the above algorithms for a simple model of pairwise RNA structural evolution; in particular, the algorithms for maximum likelihood (ML) alignment of three known RNA structures and a known phylogeny and inference of the common ancestral structure. We compared this ML algorithm to a variety of related, but simpler, techniques, including ML alignment algorithms for simpler models that omitted various aspects of the full model and also a posterior-decoding alignment algorithm for one of the simpler models. In our tests, incorporation of basepair structure was the most important factor for accurate alignment inference; appropriate use of posterior-decoding was next; and fine details of the model were least important. Posterior-decoding heuristics can be substantially faster than exact phylogenetic inference, so this motivates the use of sum-over-pairs heuristics where possible (and approximate sum-over-pairs). For more exact probabilistic inference, we discuss the use of transducer composition for ML (or MCMC) inference on phylogenies, including possible ways to make the core operations tractable.

## Author Summary

A number of leading methods for bioinformatics analysis of structural RNAs use probabilistic grammars as models for pairs of homologous RNAs. We show that any such pairwise grammar can be extended to an entire phylogeny by treating the pairwise grammar as a machine (a “transducer”) that models a single ancestor-descendant relationship in the tree, transforming one RNA structure into another. In addition to phylogenetic enhancement of current applications, such as RNA genefinding, homology detection, alignment and secondary structure prediction, this should enable probabilistic phylogenetic reconstruction of RNA sequences that are ancestral to present-day genes. We describe statistical inference algorithms, software implementations, and a simulation-based comparison of three-taxon maximum likelihood alignment to several other methods for aligning three sibling RNAs. In the Discussion we consider how the three-taxon RNA alignment-reconstruction-folding algorithm, which is currently very computationally-expensive, might be made more efficient so that larger phylogenies could be considered.

## Introduction

In 1968, Francis Crick hypothesized that the first ribosome consisted entirely of RNA, without any protein cofactors [1]. A domain structure for this primeval ribosome was recently proposed [2]. To synthesize such a reconstructed ribosome or reconstructions of other evolutionarily significant RNAs such as group II introns [3] or telomerase [4], it will be necessary to develop methods that can predict the sequences and structures of ancient RNAs based on the divergent sequences of their many descendants.

An inspection of RNA alignments, such as those in the RFAM database [5], suggests that an evolutionary model for RNA structure must eventually include multiple layers of detail: point substitutions, covariant substitutions of base-pairs [6],[7], indels [8], local changes in secondary structure such as helix slippage [9], and changes in domain structure [2]. Stochastic context-free grammars (SCFGs), which can efficiently detect the long-range correlations of RNA base-pairing structures, are natural probabilistic models of such phenomena and have been used for ncRNA homology detection [10]–[13], gene prediction [14],[15], folding [16],[17] and alignment [18]–[20].

By analogy with models of substitution processes, which are well-understood [21], we may take the problem of building phylogenetic models of RNA evolution and split it into two halves. The first half is the development of a **pairwise model**, describing the probability distribution 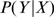 of a descendant (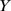) conditional on its immediate ancestor (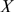). In substitution processes, the pairwise model is a conditional substitution matrix. Often (but not always) the pairwise model, representing a finite evolutionary time 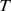, is derived from an instantaneous model of change over an infinitesimal time interval, i.e., a continuous-time Markov chain (parametrized by a rate matrix). Obtaining the transition probabilities of this chain (via exponentiation of the rate matrix) yields a pairwise model whose parameters are smoothly-varying functions of 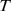. A pairwise model represents an individual branch of a phylogenetic tree, with 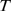 representing the length of that branch.

The second half of the phylogenetic modeling problem involves extending the model (and related inference algorithms) from a single branch to a complete phylogeny, i.e., from a pairwise model of two sequences to a **multiple-sequence model** of many sequences. In a typical situation, the sequences at the leaves of the tree are observed but those at internal nodes are not. Questions of interest then include:

- What is the likelihood for the observed sequence data?
- Can we sample (find the mode, take moments, etc.) from the posterior distribution of the unobserved sequence at the root node?
- Can we sample from the posterior of the unobserved sequences at the other internal nodes?
- Can we estimate summaries of the evolutionary history, such as the number of substitution events on each branch (for a substitution model), the alignment (for a model which includes indels), or changes in the underlying structure (for a model of RNA structure)?

For substitution models, there has been extensive work focused on answering each of these questions. Given a pairwise substitution model, questions A and B can be answered exactly by Felsenstein's pruning algorithm [22] and question C can be answered by the peeling algorithm (first presented for pedigree analysis by Elston and Stewart [23]). The estimation of evolutionary histories (question D) has been addressed by exact summarization [24] and sampling [25] approaches. Another representation of answers A–C is that the pruning and peeling algorithms (combined) are just the sum-product algorithm on a directed graphical model [26], yielding exact marginal distributions for unobserved variables. Graphical models also suggest general-purpose sampling approaches in addition to the exact sum-product algorithm.

The two halves of the reconstruction problem — developing a pairwise model and then extending it to multiple sequences — are largely independent. Felsenstein's pruning algorithm, for example, is essentially blind to the parametric form of the pairwise substitution model; it just assumes that a substitution matrix is provided for every branch. Subsequent models developed by other researchers can be plugged into the pruning algorithm without modification [27],[28].

We therefore addressed the problem of modeling the indel-evolution of multiple structured RNAs in a similarly-modular fashion by separating the creation of pairwise and multiple-sequence models. In previous work, we addressed the first (pairwise) part of the RNA reconstruction problem by describing a simple continuous-time model of RNA structural evolution [29]. This model corresponded to a Pair SCFG with a time-dependent parametrization which we used to simultaneously align and predict the structure of pairs of related RNAs. The focus of the present work is to solve the second (multiple-sequence) part of the RNA reconstruction problem by giving a general procedure for extending a pairwise model to multiple sequences related by a phylogenetic tree. This process yields a multiple-sequence SCFG, a natural model of the evolutionary relationships between multiple structured RNAs.

The main contributions of this paper are (1) an algorithm that transforms a phylogenetic ensemble of pair grammars, representing models on branches of a phylogenetic tree, into a coherent, multiple-sequence SCFG, (2) dynamic programming (DP) algorithms for performing inference under this multiple-sequence SCFG, and (3) freely-available software implementing algorithms (1) and (2) for the simplified case of a three-taxon star-topology tree. While the idea of composing conditionally-normalized models on trees is intuitive, the resulting models can be very complex, even for simple models of RNA evolution, making (1) necessary. Studies of related indel models have suggested that an implementation of dynamic programming (DP) algorithms on a three-taxon tree is sufficient to draw samples from the posterior distribution of ancestral sequences on more complex tree topologies, using Markov Chain Monte Carlo or MCMC [30]–[32], suggesting that (2) and (3) are, in principle, sufficient for analyzing trees relating many sequences.

We show that our algorithm produces a multiple-sequence grammar which is much more compact than suggested by naive approaches to model construction. We provide analyses of the asymptotic complexities of models constructed using our procedure and provide estimates of the time and memory required to reconstruct the structures of several RNA families for the case of a three-taxon phylogeny, which we have implemented in the program Indiegram. While by these estimates only the smallest sequences currently fit into affordable memory, thereby preventing us from applying our method to many problems of interest, a simulation study suggests that we can hope to accurately reconstruct ancestral structures over long evolutionary time, even in the presence of structural divergence.

In the Discussion, we speculate on algorithmic extensions that may reduce memory requirements, inspired by related work in reconstructing DNA and protein sequences.

## Methods

We describe below a general method for constructing a multiple-sequence stochastic grammar for alignment, folding and ancestral reconstruction of RNA, given a phylogenetic tree and a description of the evolutionary process acting along each branch.

### Overview

Our problem statement is this: **Given a phylogenetic tree relating several structured RNAs and a description of the evolution of a structured RNA along a single branch of the tree (in the form of a Pair SCFG), (1) find the corresponding phylogenetic multiple-sequence grammar and (2) use that grammar to reconstruct,** ***a posteriori*** **, the evolutionary histories of the RNAs**. We assume here that the phylogeny, including both the tree topology and branch lengths, is given.

This paper focuses on model construction and inference algorithms rather than the heuristics which will be necessary to make these algorithms fast enough for analysis of many biological datasets. As discussed below, the complexity of general inference algorithms is prohibitively high for many problems of interest. However, this complexity can be significantly reduced by incorporating outside knowledge. For example, if we know the consensus structure of several sequences or their individual structures, then we can constrain our algorithms accordingly. Similarly, we might consider only ancestral structures which are compatible with a given multiple sequence alignment, or a relatively small set of candidate alignments (as in the ORTHEUS program [33]). Such constraints are commonly used by programs for SCFG-based RNA sequence analysis such as QRNA [34], Stemloc [18] and CONSAN [19]. Alignment and structural constraints can be combined [18].

In the following sections we introduce more precise definitions for two-sequence models of RNA structure and outline our algorithms for (1) combining these two-sequence models on a phylogenetic tree and (2) using the composite phylogenetic grammars for inference.

### Two-sequence models

We discuss the general problem of creating state-space models of the evolution of related sequences, beginning with models of substitution processes acting at independent sites (as studied in likelihood phylogenetics) and generalizing to models of indels, first in primary sequences and then in sequences with conserved secondary structure.

A stochastic model for the evolution of one sequence (the ancestor, 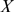) into another (the descendant, 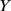) over an interval of time (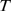) can be described by a joint distribution, 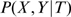. This joint distribution can be factored, 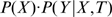, where 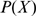 is the marginal distribution over ancestral sequences and 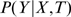 is the conditional distribution over descendant sequences given an ancestral sequence. In terms of phylogenetics, the conditional distribution 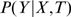 describes the evolution 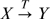 along a branch of length 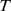.

It is possible to “multiply” two such models together. More precisely, one multiplies two conditional distributions and sums out the intermediate sequence. Thus, successive evolution along two branches 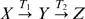 is modeled by the distribution
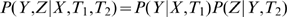
and we can sum sequence 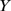 out of this, obtaining the distribution
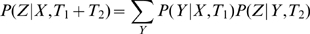
for the composite branch 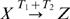.

This formalism underlies likelihood phylogenetics. Working under the independent-sites assumption, 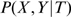 is the 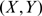
'th element of the joint substitution matrix for a single site and 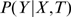 is the corresponding element of the conditional matrix. The conditional matrix is in fact the matrix exponential 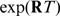, where 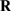 is the substitution rate matrix [24]. Composition of two branches just amounts to a matrix multiplication.

A similar formalism can be used to describe the evolution of whole sequences with indels. Suppose that the joint distribution 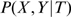 is the distribution modeled by a pair hidden Markov model (Pair HMM) [11], a probabilistic model of the evolution of two sequences under the approximation that only adjacent characters are directly correlated, and the marginal 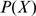 is the distribution of a single-sequence HMM, a probabilistic model of single sequences under the same approximation. The conditional distribution 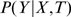 then corresponds to a conditional Pair HMM, a discrete-state machine which transforms one sequence (the input, 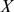) into another (the output, 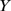). Following computational linguists, we call this conditionally-normalized state machine a **string transducer** or simply a **transducer** [35]. Because of its conditional normalization, this state machine is distinct from a standard Pair HMM. A Pair HMM has two outputs 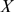 and 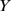 and emits symbols to both of those outputs, while a transducer absorbs symbols from the input 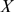 and emits symbols to the output 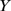. Despite this distinction, Pair HMMs and transducers share very similar inference algorithms; for example, 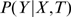 is computed using a direct analogue of the Forward algorithm [11].

We extend this formalism to the case of structured RNA as follows. Let 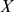 and 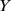 now represent structured RNA sequences or, more precisely, parse trees. A single-sequence SCFG models the marginal 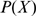; a jointly-normalized Pair SCFG [11] models the the joint distribution 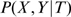. The conditional distribution 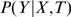 is modeled by a conditionally-normalized Pair SCFG. Following terminology from computational linguistics [36], we call this conditionally-normalized grammar a **parse-tree transducer**.

String transducers are special cases of parse-tree transducers, just as HMMs are special cases of SCFGs. Henceforth, we will drop the distinction between strings and parse trees. We will also refer interchangeably to “states” (in the state-machine representation) and “nonterminals” (in the grammar representation). Likewise, we will refer interchangeably to “state paths” (machines) and “parse trees” (grammars).

#### Terminology and normalization

Consider the stochastic grammar which generates parse trees from the marginal distribution 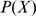. It is convenient to represent this grammar as a transducer whose input is constrained to be null, i.e. a machine that accepts a dummy (empty) input sequence, and outputs sequence 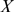. We refer to this as the **singlet transducer**. In contrast, the more general type of transducer that absorbs parse trees 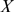 and generates modified parse trees 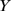 from the conditional distribution 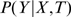 is a **branch transducer**. By definition, singlet transducers only emit symbols to their output sequence, and use a restricted set of state types. Branch transducers, in contrast, can both emit symbols to their outputs and absorb symbols from their inputs, and so use the full range of state types.

Transducers can have states of type 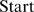, 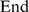, 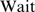, 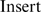 and 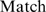. The first three state types, 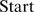, 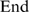 and 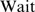, are null: they do not emit or absorb any symbols and are required solely for organizational purposes (see following section). Two types of states can emit and/or absorb symbols, 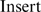 and 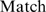. An 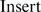 state emits a symbol to the output without absorbing anything. A 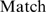 state absorbs a symbol on the input and either emits the same symbol to the output, substitutes a different output symbol, or emits no output symbol at all, the last corresponding to a deletion.

As stated above, the Pair SCFG must be conditionally normalized so that models can be chained together, extending the pairwise model to multiple sequences. The transformation rules are partitioned into co-normalized groups; within each group, the rule probabilities must sum to one. In a jointly-normalized Pair SCFG, each group corresponds to the set of all rules that can be applied to a given nonterminal (i.e., all outgoing transitions from a particular state). In a conditionally-normalized Pair SCFG, in contrast, each co-normalized group includes all rules that can be applied to a given nonterminal *for a given set of absorbed symbols*.

### Multiple-sequence models

We can use the concepts of factoring probability distributions introduced in the two-sequence framework to model the common descent of many homologous sequences. Given a phylogenetic tree and a two-sequence model, we wish to obtain a multiple-sequence SCFG describing the common descent of the observed sequences.

A singlet transducer (which emits, but does not absorb, symbols) lies at the root of the phylogeny and serves as a generative model of the ancestral sequence. To represent the evolution of an ancestral sequence into many descendant sequences, we place a branch transducer on each branch of the phylogeny.

Throughout this paper we frequently refer to two and three-taxon (star) phylogenies. In all cases, the sequence 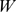 is assumed to be the (unobserved) ancestral sequence and the sequences 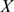, 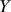, and 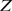 the (observed) extant sequences.

#### The composition algorithm

While this composition of conditionally-normalized models on a phylogenetic tree is intuitive, in practice building such an ensemble model is challenging due to the sheer number of possible states and transitions of the ensemble model. The maximum possible state space of the ensemble is the Cartesian product of the individual transducer state spaces. If the singlet transducer has 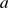 states, each branch transducer has 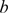 states, and the phylogeny has 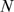 branches, then an upper bound on the number of ensemble states is 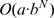. However, in practice there are many fewer states than suggested by this bound; many state configurations are not reachable. For example, for the tree with two extant sequences and a single parent, the branch transducers above leaves 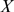 and 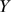 cannot simultaneously be in 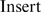 states, as this would correspond to aligning non-homologous (inserted) characters. Similarly, while an upper bound on the number of possible transitions in the transition matrix of the ensemble model is 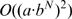, in practice models never reach this bound, due both to inaccessible configurations, such as the one described above, and the sparseness of transitions between the remaining, accessible configurations.

While the accessible state space of the ensemble is smaller than that given by the exponential upper bound, it is generally nonetheless too complex to deal with by hand. For example, the simple model of RNA structural evolution described in Results yields an ensemble model of three sequences with 230 states and 1,789 transitions. More realistic models of RNA give rise to even larger ensemble models.

We therefore need an algorithm to efficiently construct the state graph of the ensemble model, consisting of a list of accessible states and the possible transitions between them. By analogy with algorithms for uninformed graph search in artificial intelligence, the transition graph of the ensemble can be constructed by an uninformed depth-first search, where at each step of the search we obtain the next possible ensemble states by changing the state of one or more of the singlet or branch transducers. Beginning with the entire ensemble in state 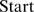, the depth-first search of states continues until all nodes are in state 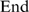.

The allowed transitions of the ensemble can be categorized as follows:

**Null Transition:** A branch transducer makes a transition into a 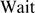 state, with no terminal emission or bifurcation.
**Terminal Emission:** A singlet or branch transducer makes a transition into a state of type 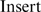, emitting left and/or right terminal symbols (e.g., a single base or base-pair). These symbols are absorbed by the immediately-descended transducers, which are pushed into states of type 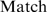 and may themselves emit terminal symbols that will be absorbed by *their* descendant transducers. This continues down the tree: The terminal symbols are passed from parents to children to grandchildren (albeit possibly being replaced by other terminal symbols as they are propagated down) and they propel branch transducers into 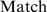 states as they go. Eventually, the cascade of emitted terminal symbols stops when all the symbols have been deleted or when the cascade reaches the leaves of the tree.
**Bifurcation:** A singlet or branch transducer makes a transition into a state of type 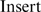 that spawns left and/or right nonterminal states. These nonterminals are processed recursively down the tree, just as in a terminal emission (conceptually, a bifurcation is a “nonterminal emission”). As with terminal emissions, absorption of nonterminal emissions propels descendant transducers into 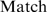 states, making transitions which may themselves propagate nonterminals further down the tree. A biologically-relevant example of a bifurcation is the insertion of a stem into an ancestral RNA structure, which may then be conserved or deleted in the descendant structures.
**End Transition:** The singlet transducer at the root makes a transition to the 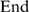 state, pushing all the descendant branch transducers into 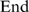 states and terminating the current branch of the parse tree.

Co-ordination between the various branch machines is achieved by specifying an ordering on the nodes and by having branch transducers pause in 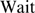 states while waiting to absorb a symbol from the node above. Only one transducer is allowed to make a spontaneous transition at a time. If this transition corresponds to a terminal emission or a bifurcation, then this may force descendant transducers into making reactive transitions.

The four types of allowed transitions listed above can be formalized as follows. Let the total order on the nodes correspond to any preorder traversal of the tree; thus, “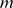 is ancestral to 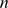
” is sufficient-but-not-necessary for “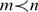.” Let 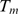 denote the singlet or branch transducer which emits symbols to node 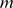. Transducer 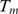 changes state if and only if one of the following three mutually-exclusive conditions holds:

Type 1: Transducer 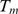 is not in a 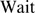 state, while all its successor transducers 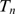 are in 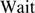 states (where 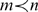). 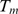 is free to make any transition.
Type 2: Transducer 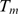 is in a 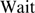 state. Its parent transducer enters a 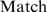 or 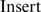 state, emitting a symbol and forcing 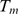 into a 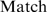 state so it can absorb that symbol.
Type 3: Transducer 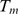 is in a 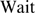 state. Its parent transducer enters the 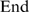 state, forcing 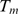 into the 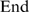 state as well.

A notational prescription for the allowed transitions may be found in Text S1.

#### How the ensemble generates multiple alignments

The possible transitions of the ensemble generate multiple alignments as follows:

1. The singlet transducer and all branch transducers begin in their respective 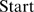 states.
2. Before any residues can appear at the root, the branch transducers all wind back into 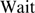 states, via type-1 transitions. This occurs in reverse order (i.e., a postorder traversal of the tree).
3. During this initial windback, clade-specific insertions can occur. This process is described in detail at step 9.
4. With all the branch transducers wound back into 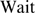 states, the singlet transducer makes a (type-1) transition into an 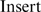 state, emitting a symbol to the sequence at the root node.
5. The transducers on outgoing branches from the root then make (type-2) transitions into 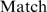 states, either copying the root symbol to their own outputs, substituting it for a different symbol or staying silent (this silence corresponds to a clade-specific deletion; in our formalism, both substitutions and deletions are handled by 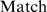 states.)
6. The transducers on branches one step away from the root then process the symbols which reached them (if any did), followed by transducers on branches two steps away from the root, then three steps, and so on (these can all be regarded as occurring simultaneously, in a single cascading wave of emissions).
7. Eventually the emitted symbols are propagated, via type-2 transitions, all the way to the tips of the tree (if they survived) or to the nodes where they were deleted (if they did not survive). The wave of type-2 transitions has left a lot of branch transducers in 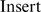 and 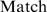 states.
8. The branch transducers then, in postorder, each wind back into 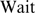 states, just as at step 2. (These windback transitions can be collapsed into a single ensemble transition, as with the emission cascade; however, the windback may be interrupted by clade-specific insertions; see below.)
9. During the postorder windback, each branch transducer gets an opportunity to generate a new symbol (via type-1 transitions to 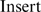 states). (If such a transition to 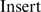 occurs, it corresponds to a clade-specific insertion. This insertion is propagated down the tree via a wave of type-2 transitions, as above, then we go back to step 7.)
10. Eventually, the entire ensemble has wound back, so that every transducer is in a 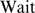 state except the singlet transducer at the root, which is still in an 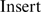 state. At this point, all clade-specific insertions have been processed.
11. The singlet transducer now makes another type-1 transition. If this transition is to an 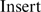 state, the entire cycle begins again: the singlet transducer emits the next symbol at the root, and we go back to step 4.
12. If, on the other hand, the singlet transducer enters its 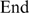 state, then a wave of type-3 transitions drives all the branch transducers into their respective 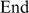 states too, bringing the entire ensemble to a halt.

#### Complexity of the transducer ensemble

The total sizes of both the state space and the transition matrix are, in general, dramatically smaller than implied by the exponential upper bounds of 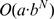 and 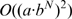. While we do not have provable bounds on the size of the state space, we have observed that the size of the state space is roughly linear in the number of branches, 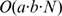, and the number of transitions is approximately linear in the number of states for several pairwise models, including the pairwise model which we use here. However, these empirical observations are based on a limited class of pairwise models and we do not have theoretical results for how they will generalize to other pairwise models. We do believe, however, that the worst-case exponential bound will be avoided by (1) omitting inaccessible state configurations and (2) eliminating null windback states as described in the following section (which we believe will prevent affine gap penalties from generating exponential growth in the number of states).

Therefore, for the models which we have characterized, the search algorithm given above for enumerating all allowed transitions of the ensemble model typically generates 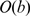 transitions from any given state, thereby creating a very sparse transition matrix of size 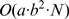.

### Inference algorithms for multiple-sequence models

In this section, we describe dynamic programming (DP) algorithms for inferring the alignment, structure and evolutionary history of multiple related RNAs, using the multiple-sequence SCFG we have derived.

The transducer composition algorithm described above constructs a phylogenetic SCFG for both ancestral and extant sequences. A parse tree for this SCFG represents a structural and evolutionary explanation of the extant sequences, including a complete ancestral reconstruction. Consequently, given a set of extant sequences, many of the questions of interest to us can be reduced to searches over, or summarizations of, the set of possible parse trees.

Well-known algorithms already exist for maxing or summing over SCFG parse tree likelihoods. The Cocke-Younger-Kasami (CYK) algorithm performs maximum-likelihood (ML) inference; the Inside algorithm can be used to sum over parse trees or sample them *a posteriori*; and the Inside-Outside algorithm yields posterior probabilities for individual parse tree nodes [11].

All of these algorithms are, however, complicated (at least in our models) by the existence of “null cycles” in the grammar. A null cycle is a parse tree fragment that is redundant and could be removed, such as a detour through 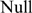 states (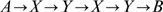) that could be replaced by a direct transition (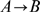). Biologically, null cycles correspond to fragments of ancestral sequence that were universally deleted and therefore are unobserved in any of the extant sequences. These unobserved fragments can be unbounded in length (and so, therefore, can the parse tree). Within the CYK, Inside and Outside recursions, this causes cyclic dependencies which cannot be resolved.

Below we describe a method to eliminate null cycles from the ensemble model by transforming any SCFG to an equivalent acyclic SCFG. We then present multiple-sequence versions of the CYK, Inside and Outside algorithms.

While some sort of null-cycle elimination is often required in order to deal with cyclic dependencies, there are several ways to accomplish this other than the algorithm presented below. A simpler approach (that only works for the CYK algorithm) appears in the computational linguistics literature [37]. We have also developed a heuristic for CYK that simply ignores null cycles as well as an iterative approximation that loops several times over cyclically-dependent cells of the DP matrix until the estimate starts to converge. For conciseness, we have omitted descriptions of these methods, presenting only the exact elimination algorithm.

#### Exact elimination of null cycles in SCFGs

As noted above, the ensemble grammar contains many rules that can be applied redundantly, together or in isolation, to generate subtrees of the parse tree that do not generate any terminals. This generates cyclic dependencies in the standard DP recursions for inference. In this subsection, we describe how to transform the SCFG so as to eliminate such redundant rules, yielding strictly acyclic DP recursions. This transformation can be applied to any SCFG so as to remove null states and/or bifurcations: the procedure is not restricted to grammars that were generated using our transducer composition algorithm.

We begin by identifying two distinct classes of redundant parse-subtree: **empty bifurcations** and **empty paths**. We will eliminate each of these in turn.

An **empty bifurcation** occurs when a child branch of a bifurcation state transitions to the 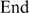 state without emitting any symbols and can be removed from the model by creating an effective direct transition encapsulating the empty bifurcation. For example, we can create an effective direct transition 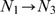 between null states 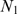 and 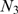 in place of the empty parse-subtree 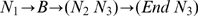, where 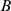 is a bifurcation state with children 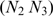. Bifurcation states are the most computationally-costly part of our models, so it is important to eliminate as many as possible without reducing model expressiveness.

In contrast, an **empty path** is defined as any parse-subtree *without* bifurcations that does not emit terminal symbols. If 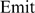 states 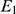 and 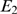 are connected in the state graph via 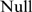 states 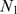 and 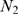, then the path 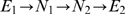 with probability 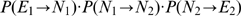 can be replaced by a single direct transition 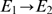 with an identical probability.

Empty paths occur in Hidden Markov Models (which are special cases of SCFGs) and independent-sites models (which can be viewed as special cases of HMMs). Conceptually, empty paths can represent histories that are valid according to the model but cannot be resolved by direct observation. Such null events can be real (e.g., ancestral residues that have been deleted in all extant lineages) or they can be artefactual (e.g., transitions between placeholder null states of an HMM).

In our composite model, empty paths occur whenever a series of branch transducers winds back into 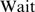 states. Empty bifurcations occur when an entire substructure, present in an ancestor, is deleted in all that ancestor's extant descendants.

Empty paths and empty bifurcations are problematic because they can be combined to give finite-probability sequences of rules that transform a nonterminal back into itself, with no observable emissions. We refer to such sequences of rules as **null cycles**. As noted, null cycles generate cyclic dependencies in the CYK, Inside & Outside algorithms. Our goal is an algorithmic procedure to resolve these dependencies and account for the likelihood of such cycles by exact marginalization.

For simpler models, solutions to this problem are published. Missing (empty) columns in independent-sites models can be accounted for by applying a correction factor 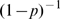 to account for the proportion of columns 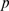 that are unobserved [38]. The slightly more complicated situation of missing emissions in a HMM can be dealt with by summing over all empty paths, yielding a geometric series that is solvable by matrix inversion [39]–[41]. Such algorithms effectively replace the HMM with another HMM that contains no null cycles but is equivalent to the original, in that it models the same probability distribution over sequences. However, these solutions do not easily generalize to SCFGs (which may have empty bifurcations as well as empty paths).

Text S2 includes a complete formal algorithm for exact null-cycle elimination in SCFGs, along with procedures for probabilistically restoring null cycles to sampled parse trees and Inside-Outside expectation counts. Informally, the essence of the algorithm is contained within the following two steps:

- separating bifurcations into those which have one or more empty children (and can therefore be represented using transition or termination rules) and those that have two nonempty children;
- replacing all empty paths through null states with effective direct transitions between non-null states, obtaining sum-over-paths probabilities by inverting the grammar's transition matrix.

Note that step (i) is unique to SCFGs; step (ii), in contrast, is very similar to the empty-path elimination algorithm for HMMs.

#### Dynamic programming algorithms for inference

Once we have performed the transformations described above to remove null cycles from the multiple-sequence SCFGs generated by our model-construction algorithm, we can compute likelihoods and sample parse trees using the standard CYK, Inside and Outside algorithms for multiple-sequence SCFGs [11],[42].

The asymptotic time and memory complexities of our inference algorithms are essentially the same as for Sankoff's algorithm [42]: the DP algorithms take memory 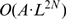 and time 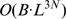 for 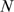 sequences of length 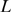, where 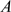 is the number of (accessible) states in the multiple-SCFG and 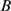 is the number of bifurcations. Note that 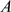 and 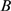 are also dependent on 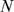 (see “The TKFST model on a three-taxon phylogeny”).

Exact inference on a star phylogeny with 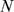 extant sequences therefore has complexities 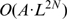 and 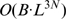 in memory and time (respectively) for a multiple-SCFG with 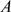 states and 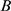 bifurcations. As described earlier, in practice we frequently have expert knowledge (such as a curated multiple alignment) about the structures and/or evolutionary histories of the sequences of interest. We can use this knowledge as a constraint to reduce the accessible volume, and hence the storage requirements, of the DP matrix [18]. The Inside, Outside, and CYK+traceback algorithms for a three-taxon star phylogeny can be constrained using the “fold envelope” concept, which will now be described.

We use the fold envelope concept [29],[43] to constrain the set of structures which our algorithms consider. A fold envelope 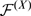 for a sequence 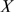 is a set of coordinate pairs satisfying
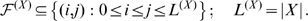
(1)We consider a subsequence 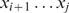 only if the corresponding coordinate pair 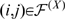. The unconstrained fold envelope has set equality in Equation 1.

An 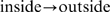 ordering is used for the iteration in the Inside algorithm: Subsequences are ordered such that each successive subsequence contains all previous subsequences in the fold envelope. More precisely, subsequences in 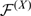 are sorted in the same order as coordinate pairs 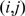 are generated by the iteration 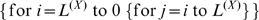.

The Outside algorithm uses the exact reverse of the 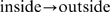 ordering described above; we call this the 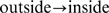 ordering. Subsequences in 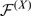 are sorted in the same order as coordinate pairs 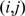 are generated by the iteration 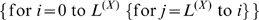.

We frequently refer to subsequences by their index in the fold envelope. The 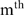 subsequence in 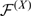 is labeled 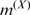 and corresponds to the coordinate pair 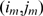. The index of a pair 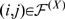 is 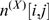.

In order to take full advantage of the reduction in computational complexity offered by restricting our inference algorithms to subsequences contained in the fold envelopes, we must avoid iterating over unreachable combinations of subsequences (unreachable because they are not permitted by the fold envelope constraints). An efficient implementation relies on iterators over subsequences in the fold envelope which are connected by production rules of the ensemble grammar. Inward and outward emission connections for a sequence 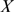, specifying which subsequence is reachable from a given subsequence 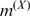 and ensemble state ***b***, are defined as
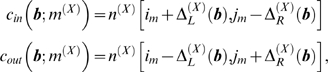
where the quantities 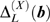 and 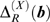 are the lengths of the left and right emissions of the ensemble state ***b*** to the sequence *X*. (Recall that the m^th^ subsequence in 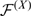 is labeled *m* ^(*X*)^ and corresponds to the coordinate pair (*i_m_*, *j_m_*).) The emission connection is undefined if the corresponding subsequence is not in the fold envelope. Inward, outward-left and outward-right bifurcation connections, specifying which subsequences are connected by bifurcation production rules of the ensemble SCFG, are defined for a subsequence *n* ^(*X*)^ as
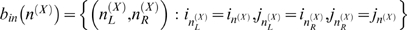
(2)

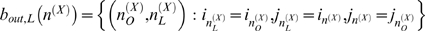
(3)

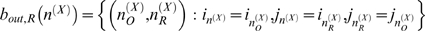
(4)We generally write out explicit subsequence coordinate pairs (*i*, *j*) when their usage will make mathematical formulas clearer and fold-envelope labels *n* ^(*X*)^ when writing pseudocode.

Using the fold envelope formalism, the main iteration over cells in the Inside and CYK matrices can be expressed as three nested loops: one for each sequence, traversing the fold envelope subsequences in inside→outside order. Conversely, the main iteration of the Outside algorithm consists of three nested outside→inside loops.

The Inside algorithm is used to calculate the likelihood of sequences under an ensemble model. It is analogous to the Forward algorithm for HMMs.

**Figure 1:**
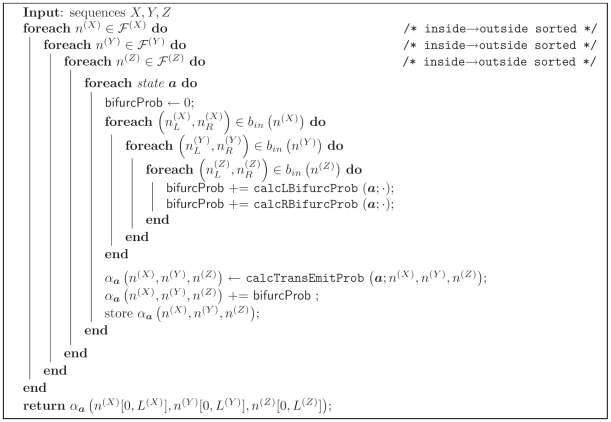
Algorithm 1. The constrained Inside algorithm for three sequences *X*, *Y*, *Z*. Ensemble states 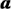 in the iteration over states are sorted in Inside fill order with Emit states first, then Null states in reverse topological order.

The inside probability 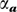
(*n* ^(*X*)^, *n* ^(*Y*)^, *n* ^(*Z*)^) is the summed probability of the triplet of subsequences (*n* ^(*X*)^, *n* ^(*Y*)^, *n* ^(*Z*)^) for sequences *X*,*Y*,*Z* under all paths through the model which are rooted in state 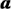. Figure 1 gives pseudocode for the fold-envelope version of the Inside algorithm. The subroutines calcTransEmitProb, calcLBifurcProb and calcRBifurcProb used in the Inside algorithm are defined below.

The transition and emission probability calcTransEmitProb(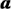; ·) can be calculated by iterating over ensemble states ***b*** which connect the subsequence triplet (*n* ^(*X*)^, *n* ^(*Y*)^, *n* ^(*Z*)^) to others in the fold envelopes.

**Figure 2:**
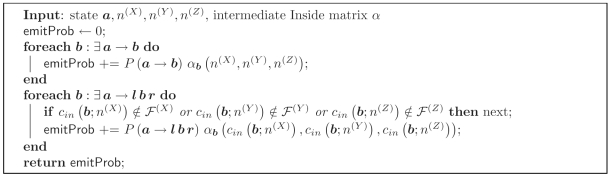
Algorithm 2. Subroutine calcTransEmitProb() for the Inside algorithm. 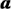 and *b* are ensemble states; *l* and *r* are left and right terminal emissions.

Pseudocode for the constrained calculation is given in Figure 2.

The left-bifurcation probability for an ensemble state 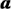 bifurcating to two ensemble states, calcLBifurcProb (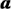; 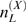, 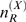, 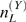, 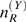, 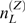, 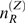), is
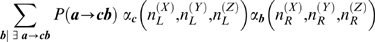
and the right-bifurcation probability for an ensemble state 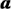 bifurcating to two ensemble states, calcRBifurcProb (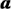; 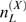, 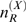, 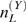, 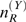, 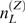, 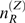), is
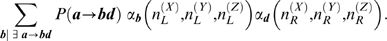
The boundary condition of the probability of 0-length subsequences is determined by the probability of transitions to End. The termination condition is
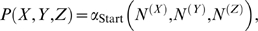
where Start is the unique start state of the ensemble grammar and *N* ^(*X*)^ is the outermost subsequence for sequence *X*, etc.

Note that we are assuming that the transformations described in “Exact elimination of null cycles in SCFGs” have been performed, such that there are no cycles of Null states as well as no empty bifurcations.

The CYK algorithm is used to calculate the probability of the most-likely state path (or parse) capable of generating the input sequences. It is analogous to the Viterbi algorithm for HMMs.

The CYK algorithm can be obtained from the Inside algorithm by replacing sums over paths through the ensemble model with the max operation. The CYK probability for indices 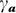
(*n* ^(*X*)^, *n* ^(*Y*)^, *n* ^(*Z*)^) then represents the probability of the most likely path through the model generating the triplet of subsequences (*n* ^(*X*)^, *n* ^(*Y*)^, *n* ^(*Z*)^).

**Figure 3:**
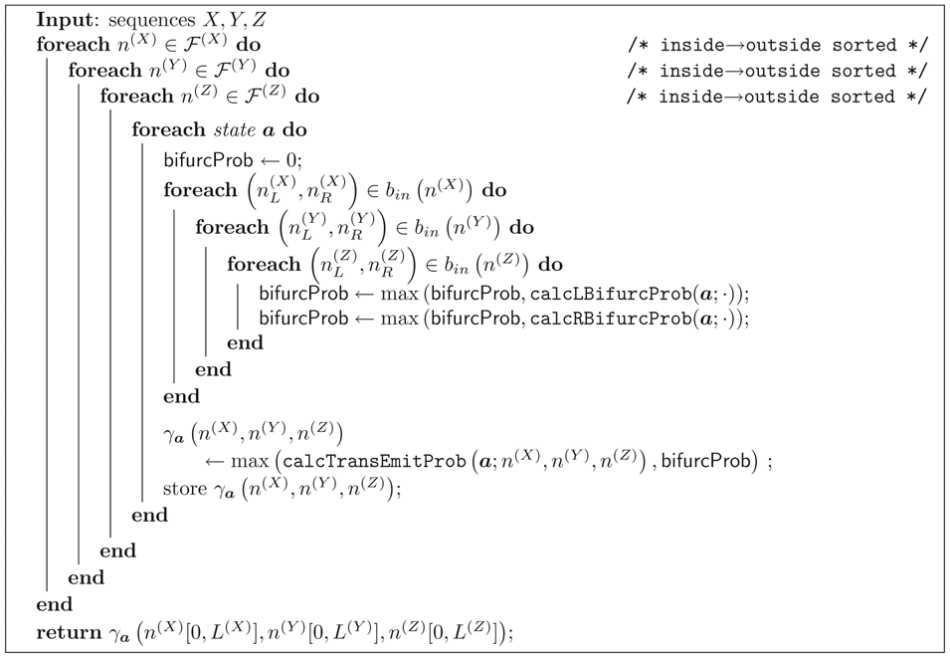
Algorithm 3. The constrained CYK algorithm for three sequences *X*, *Y*, *Z*. Ensemble states 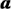 in the iteration over states are sorted in Inside fill order with Emit states first, then Null states in reverse topological order.

**Figure 4:**
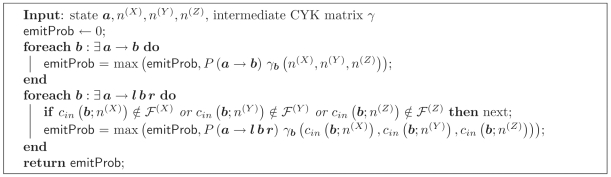
Algorithm 4. Subroutine calcTransEmitProb() for the CYK algorithm. 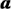 and *b* are ensemble states; *l* and *r* are left and right terminal emissions.

The resulting CYK algorithm is shown in Figure 3. The subroutine caleTransEmitProb is defined in Figure 4. The subroutines calcLBifurcProb and calcRBifurcProb used in the CYK algorithm are defined as
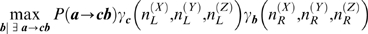
and
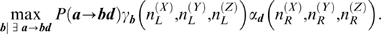



**Figure 5:**
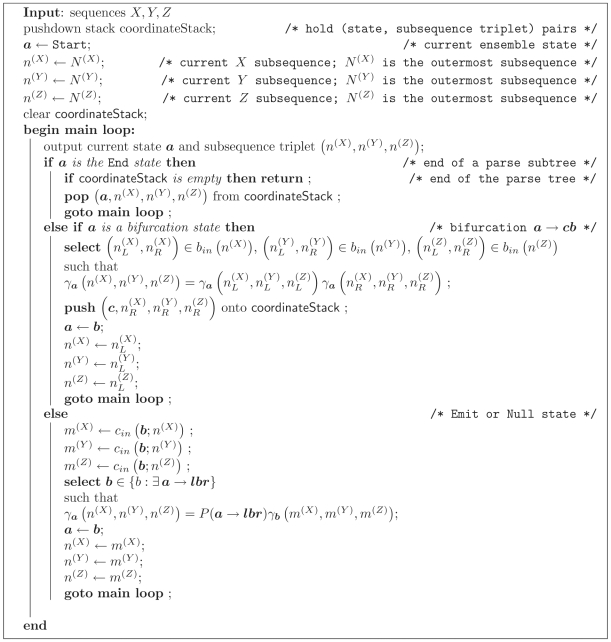
Algorithm 5. The constrained CYK traceback algorithm for three sequences *X*, *Y*, *Z*.

The CYK traceback algorithm, in combination with the CYK algorithm, is used to find the most-likely state path generating the extant sequences (in other words, the maximum-likelihood parse generating the observed data). It is analogous to the Viterbi traceback algorithm for HMMs. Figure 5 gives the constrained CYK traceback algorithm.

The Outside algorithm is primarily used an an intermediary for calculating nucleotide-level posterior probabilities, e.g. for posterior decoding on the model. It is analogous to the Backward algorithm for HMMs.

**Figure 6:**
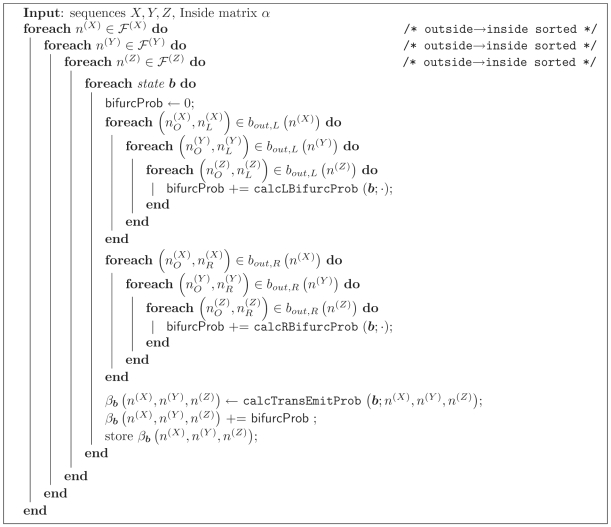
Algorithm 6. The constrained Outside algorithm for three sequences *X*,*Y*,*Z*. Ensemble states *a* in the iteration over states are sorted in Outside fill order with Emit states first, then Null states in topological order.

The outside probability *β* ***_b_*** (*n* ^(*X*)^, *n* ^(*Y*)^, *n* ^(*Z*)^) for an ensemble state ***b*** is the summed probability of the sequences *X*,*Y*,*Z* under all paths through the ensemble model which are rooted in the start state of the model, excluding all paths for the triplet of subsequences (*n* ^(*X*)^, *n* ^(*Y*)^, *n* ^(*Z*)^) which are rooted in the ensemble state ***b***. Figure 6 gives pseudocode for the fold-envelope version of the Outside algorithm. The subroutines calcTransEmitProb, calcLBifurcProb and calcRBifurcProb used in the Outside algorithm are defined below.

**Figure 7:**
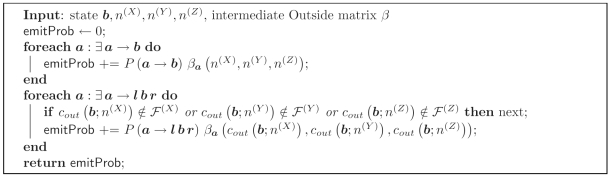
Algorithm 7. Subroutine calcTransEmitProb() for the Outside algorithm. 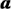 and *b* are ensemble states; *l* and *r* are left and right terminal emissions.

As with the Inside and CYK algorithms, the transition and emission probability calcTransEmitProb can be calculated efficiently using the subsequence connections defined earlier (Figure 7). The left-bifurcation probability calcLBifurcProb (***b***; 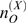, 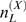, 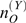, 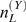, 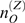, 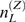) is
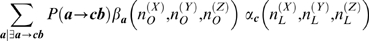
and the right-bifurcation probability calcRBifurcProb (***b***; 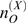, 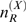, 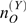, 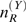, 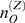, 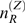) is
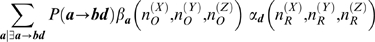
The boundary condition is just
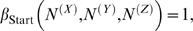
where *N* ^(*X*)^ is the outermost subsequence for sequence *X*, etc.

## Results

### Automated grammar construction

We implemented our model construction algorithm on the three-taxon star phylogeny. Given a singlet transducer modeling ancestral structures and a branch transducer modeling structural evolution, our Perl modules generate C++ code for the corresponding jointly-normalized three-sequence (Triplet) SCFG. Any model of structural evolution which can be represented as a Pair SCFG and factored into singlet and branch transducers is permitted as input to the packages, allowing for flexible, automated model design. The available software is described in Text S3.

### A simple model of RNA structural evolution

We illustrated our method for building models of structured sequences using a model which was introduced in previous work, the TKF Structure Tree [29], a simplified probabilistic model of the evolution of RNA structure.

The TKF Structure Tree (TKFST) model is based on the Thorne-Kishino-Felsenstein (TKF) model of the stochastic evolution of primary sequences via indel events [44]. In the original TKF model, sequence evolves under a time-homogeneous linear birth-death-immigration process [45]. Single characters (“links”) are inserted with rate 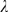 and deleted with rate 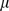. At equilibrium, sequences obey a geometric length distribution with parameter 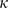. Although this model has flaws (e.g., it lacks affine gap penalties, rate heterogeneity and context-dependent mutation rates), it illustrates many of the key ideas used by more sophisticated indel models, notably the possibility for systematic derivation of pairwise alignment automata from first principles via analysis of birth-death processes [44],[46].

**Figure 8:**
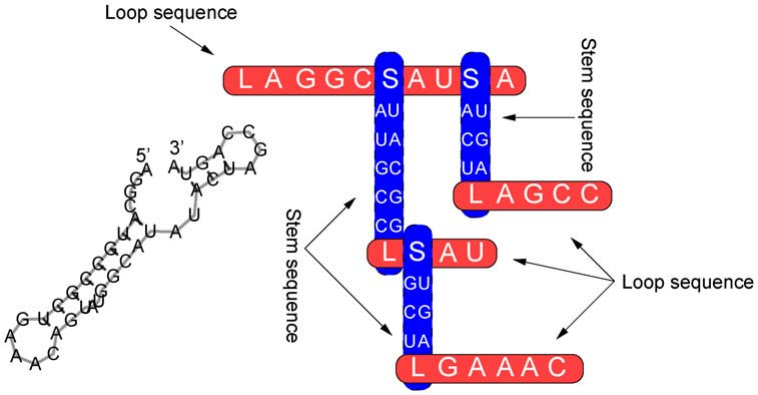
The TKF Structure Tree model represents the evolution of RNA structure as nested stem and loop sequences. The model consists of recursively nested loop sequences (gray, horizontal) and stem sequences (black, vertical). The loops are sequences of unpaired bases and the stems are sequences of covarying base-pairs. Both loop and stem sequences evolve according to the Thorne-Kishino-Felsenstein (TKF) model [44] of molecular evolution. Figure is extended from a similar version in [29].

**Figure 9:**
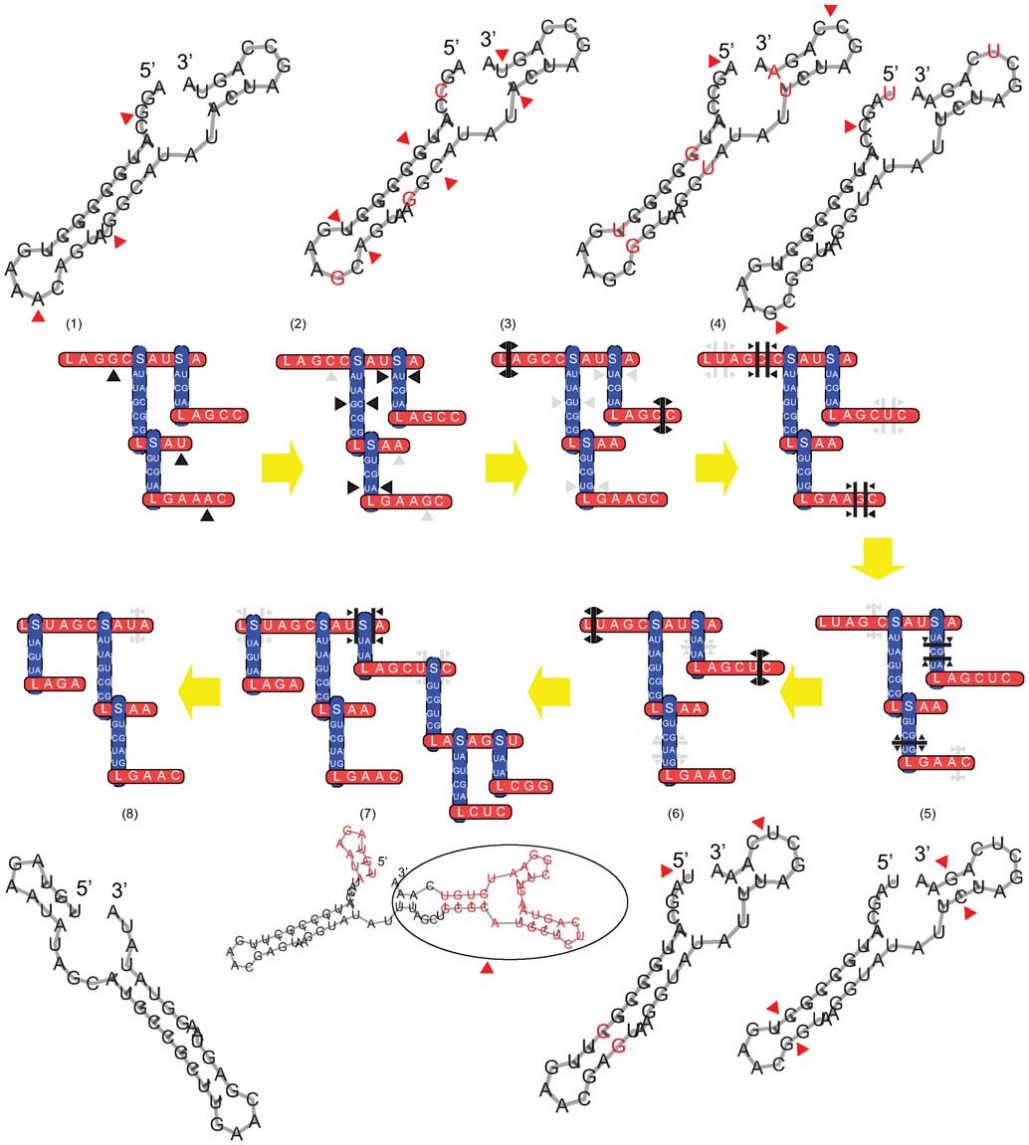
Evolution of a RNA structure under the TKF Structure Tree model. The TKF Structure Tree model includes phenomena such as point mutations in loop sequences (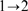 and 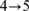), covariant mutations in stem sequences (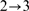), insertions in loop sequences (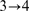), insertions in stem sequences (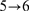), structural insertions (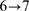), and structural deletions (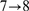). Figure is extended from a similar version in [29].

The TKF Structure Tree model is an extension of the TKF model to RNA structure. In this model, loop and stem regions are mutually nested (Figure 8): the parameter 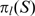 determines the proportion of links within loop sequences that are nested stems, and every stem sequence has a nested loop at the end. Single bases are inserted and deleted in loops with rates 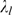 and 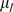; similarly, base-pairs are inserted and deleted in stems with rates 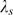 and 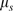. Both loops and stems have geometric length distributions with parameters 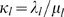 and 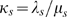. Insertions of a new stem into an existing loop sequence (or deletions of an existing stem) occur at the same rate as single-base insertions (or deletions) and can model large-scale structural changes (Figure 9).

We parametrized the singlet and branch transducers of the TKFST model using estimates reported by a phylo-grammar for RNA secondary structure prediction, PFOLD [16], and an implementation of pairwise alignment for the TKF Structure Tree model, Evoldoer [29]. The equilibrium distributions of unpaired and paired nucleotides of the singlet and branch transducers, as well as the substitution models of unpaired and paired nucleotides of the branch transducers, were derived from the substitution rate matrices of the PFOLD program. These rate matrices, which have proven useful for RNA structure prediction [16],[17],[47], were derived from the Bayreuth tRNA database [48] and the European large subunit rRNA database [49].

This continuous-time model corresponds to a Pair SCFG and as such fits neatly into our modeling framework once the probability distribution is appropriately factored into marginal and conditional distributions (generated by singlet and branch transducers). Tables 1 and 2 show the states and transitions of the singlet transducer (single-sequence SCFG) which generates ancestral sequence under the Structure Tree model. Tables 3 and 4 show the states and transitions of the branch transducer (conditionally-normalized Pair SCFG) which evolves a sequence and structure along a branch of the phylogenetic tree.

**Table 1:** State types of the singlet transducer (single-sequence SCFG) of the TKF Structure Tree model.

State	type	absorb	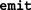	description
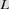	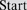			Start of a loop
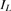	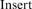		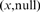	Single-base emission
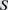	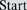			Start of a stem
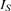	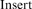		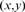	Base-pair emission
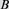	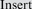		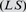	Bifurcation

Singlet transducers can only have states of type 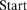 or 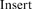.

**Table 2:** Singlet transducer (single-sequence SCFG) of the TKF Structure Tree model.

Source→Destinatioon	probability	Source→Destinatioon	probability
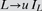	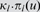	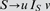	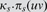
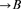	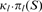	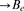	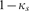
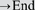	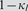		
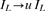	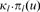	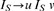	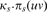
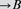	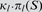	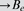	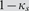
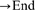	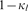		
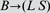	1		
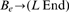	1		

The state types for this model are shown in Table 1. The singlet transducer generates ancestral RNA sequences and structures. We use the notation of formal grammars to represent state transformation rules; for example, the rule 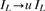 corresponds to (in a Pair HMM) an 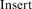 state 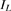 emitting a nucleotide 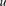 and then making a self-transition. Both loop (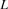 and 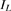) and stem (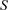 and 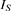) sequence evolve as TKF sequences with length parameters 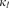 and 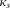 (defined in “A simple model of RNA structural evolution”). 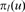 and 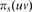 are the equilibrium distributions of unpaired nucleotides 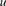 and paired nucleotides 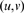 and are normalized such that 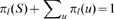 and 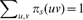. The bifurcation state 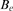 is used to end stem sequences (only loop sequences are allowed to transition to the empty string).

**Table 3:** State types of the branch transducer (conditionally-normalized Pair SCFG) of the TKF Structure Tree model.

State	type	absorb	emit	description
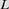	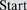			Start of a loop
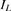	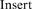		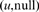	Single-base insertion
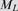	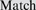	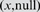	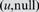	Single-base substitution
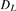	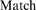	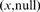		Single-base deletion
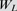	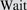			Wait for next base
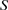	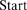			Start of a stem
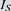	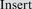		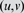	Base-pair insertion
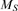	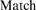	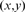	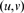	Base-pair substitution
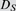	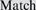	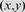		Base-pair deletion
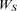	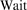			Wait for next base-pair
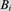	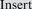		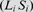	Stem insertion
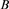	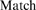	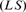	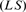	Stem conservation
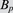	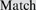	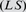	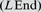	Stem deletion
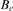	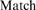	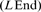	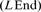	Stem extinction

States which have the same names as states of the singlet transducer in Table 1 are the branch-transducer equivalents of the corresponding singlet-transducer states (e.g., a 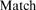 state might be the branch equivalent of an 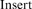 state). States 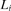 and 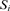 are the 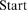 states of a sub-model (not shown) identical in structure to the singlet transducer. They are used to insert a new stem-loop structure.

**Table 4:** Branch transducer (conditionally-normalized Pair SCFG) of the TKF Structure Tree model.

Source→Destinatioon	probability	Source→Destinatioon	probability
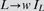	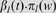	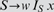	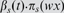
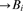	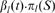	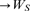	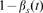
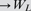	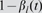		
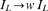	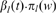	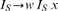	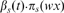
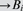	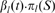	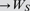	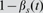
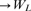	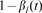		
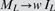	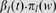	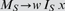	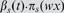
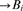	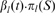	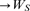	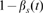
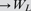	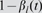		
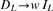	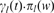	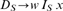	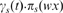
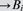	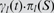	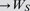	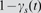
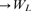	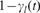		
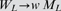	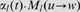	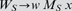	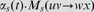
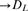	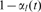	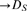	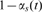
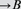	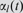	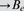	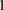
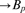	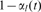		
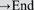	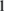		
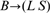	1	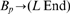	1
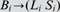	1	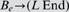	1

The state types for this model are shown in Table 3. The branch transducer evolves a sequence and structure along a branch of the phylogenetic tree. States 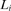 and 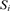 are the 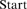 states for a sub-model corresponding to an insertion of a new stem in the descendant sequences; the sub-model (not shown) is identical in structure to the singlet transducer shown in Table 2. 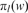 and 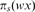 are the equilibrium distributions of, respectively, descendant unpaired nucleotide 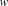 and descendant paired nucleotides 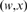; 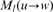 and 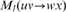 are the conditional distributions (i.e., match probabilities) of a descendant unpaired nucleotide 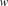 given an ancestral unpaired nucleotide 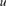 and descendant paired nucleotides 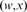 given ancestral nucleotides 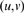. The functions 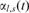, 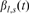 and 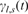 are parametrized by the insertion and deletion rates of the TKFST model and are defined in “A simple model of RNA structural evolution”.

The equilibrium distribution and transition probabilities between states of the TKFST model can be expressed in terms of functions of the evolutionary time along a branch and the insertion and deletion rates 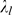 and 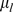 of the model. The length of ancestral sequences is geometric in 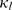 (Table 2), defined as 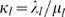. The three functions 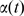, 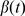 and 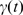 which govern the transition probabilities in Table 4 are defined for loop sequences as
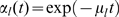


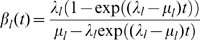


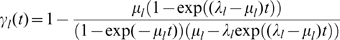
and similarly for stem sequences [29].

The above-described TKFST SCFGs must be transformed slightly before they can be loaded into Indiegram. The grammars are presented in Indiegram format in Text S4.

A few other useful statistics for the TKFST model: the expected number of links in a loop sequence is 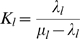 and in a stem sequence 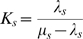. Since 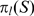 of the links in a loop sequence are nested stems, and since each stem has twice as many nucleotides as it has links (since each link is a base *pair*), the expected number of bases in a loop sequence is
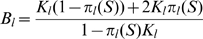
The expected number of bases in a stem sequence is
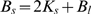
The expected number of bases that are created/removed when a loop-sequence link is inserted/deleted is
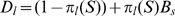
The expected number of stems directly rooted in a given loop sequence is 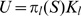 and the expected number of stems directly rooted in, **or indirectly descended from**, a given loop sequence is 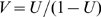 (note that this is also the expected total number of loop sequences indirectly descended from a given loop sequence). Therefore, in the equilibrium structure, the expected number of stems is 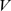; of loops, 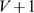; of unpaired bases, 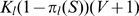; and of base-pairs, 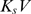. In a tree with total branch length 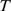, the expected number of single-base deletions is 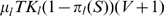; of base-pair deletions, 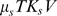; and of substructure deletions, 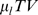.

### Assessing TKFST as a model of RNA structure

The TKFST model, like the original TKF model, probably needs refinements in order to accurately model many structural RNAs. For example, it fails to model certain phenomena observed in natural RNA structures (such as base-stacking or tetraloops) and in alignments of those structures (such as helix slippage). We assessed its appropriateness as a model of RNA structural evolution by conducting benchmarks of its capabilities for (1) multiple sequence alignment of structured RNAs, summing over all possible structures, and (2) structure prediction of homologous structured RNAs and comparing its performance to Stemloc (one of the better-performing pairwise SCFGs used for RNA multiple alignment [20]). The results of these benchmarks, reported in Table 5 and Table 6, suggest that TKFST is a useful guide for deriving more complicated models of RNA evolution: while it has relatively poor sensitivity (but high positive predictive value) as a base-pairing predictor, it is competitive with one of the most accurate RNA multiple sequence alignment programs [20].

**Table 5:** Percentage sensitivity and positive predictive value (Sensitivity/PPV) for pairwise nucleotide-level alignments in the BRalibaseII benchmark.

	U5	g2intron	rRNA	tRNA
TKFST grammar	81.6/81.7	**75.4**/**75.0**	91.4/92.6	**94.6**/**94.4**
Stemloc grammar	**82.6**/**83.7**	74.2/74.8	**92.6**/**92.8**	93.2/93.9

We compared the performance of the TKFST model for progressive multiple alignment of RNAs against the performance of a grammar with a richer model of RNA structure (Stemloc [18]). Sensitivity is defined as 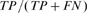 and PPV is defined as 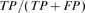, where TP is the number of true positives (correctly aligned residue pairs), FN is the number of false negatives (residue pairs that should have been aligned but were not) and FP is the number of false positives (residue pairs that were incorrectly aligned). These statistics are summed over all pairs of sequences in the multiple alignment; therefore, “Sensitivity” for pairwise residue alignments is equivalent to the Sum of Pairs Score or SPS [103]. “g2intron” is the RFAM entry Intron_gpII, containing domains V and VI of the Group II intron.

**Table 6:** Percentage sensitivity and positive predictive value (Sensitivity/PPV) for predicted base-pairs in the BRalibaseII benchmark.

	U5	g2intron	rRNA	tRNA
TKFST grammar	37.9/68.0	42.1/63.8	37.4/66.5	70.9/**88.3**
Stemloc grammar	74.9/**73.9**	64.3/56.7	51.0/59.0	74.0/76.4

We compared the performance of the TKFST model for structure-prediction accuracy during progressive multiple alignment of RNAs against the performance of a grammar with a richer model of RNA structure (Stemloc [18]). “g2intron” is the RFAM entry Intron_gpII, containing domains V and VI of the Group II intron.

TKFST's poorer performance at base-pairing prediction is likely due to its much-simpler model of RNA structure. The richer grammar, as described in [18], is much more complex than TKFST: excluding the substitution model, it has 14 free parameters (compared to TKFST's 4), uses an affine gap penalty (compared to TKFST's linear gap penalty), and explicitly models structural features such as multiple-branched loops, symmetric/asymmetric bulges, and minimum loop lengths. Unlike TKFST, the richer grammar is structurally unambiguous: a one-to-one mapping exists from structures to parse trees. Although we use the TKFST model as an illustrative example of a Pair SCFG that can be extended with our method, the model is not fundamental to our approach and can be replaced by a different and more realistic pairwise model, such as the Stemloc pairwise SCFG used in these comparisons [20]. We anticipate that further improvements should be possible by reviewing other comparisons of SCFGs at structure prediction, such as the study of [17].

### The TKFST model on a three-taxon phylogeny

We used our model-construction algorithm to build the grammar corresponding to the TKFST model acting on a star phylogeny with three (extant) leaf sequences and a single (unobserved) ancestral sequence. We chose this phylogeny for two reasons: (1) it is the simplest extension of the well-studied, standard two-sequence (Pair SCFG) model and (2) algorithms on a phylogeny with three leaves should be sufficient for ergodic sampling of reconstructions on any larger phylogeny, using, e.g., a Gibbs-sampling MCMC kernel [31] or a progressive suboptimal-alignment sampling heuristic [33].

The statistics of the TKFST model on the three-taxon phylogeny illustrate the advantages of our procedure for model construction. While the singlet and branch transducers are relatively simple—the singlet transducer, shown in Table 2, has 7 total states and 2 bifurcation states and the branch transducer, shown in Table 4, has 21 total states and 6 bifurcation states—the ensemble model of three extant sequences is very complex. The naive exponential upper-bound gives a maximal state space of size 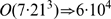 states. Using our uninformed search algorithm, we determined that there are 287 accessible states and 686 possible transitions between these states (compare with the 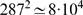 transitions estimated with the exponential calculation). After performing the transformations described in “Exact elimination of null cycles in SCFGs” to eliminate useless windback states, the ensemble model has a reduced state space with 230 states, albeit at the cost of extra transitions, bring the total to 1,789 transitions (here we are trading reduced memory complexity, which is linear in the number of states, for increased time complexity, which is linear in the number of transitions). Note that both before and after the reduction in complexity, the total number of states and transitions are less than the approximate bounds of 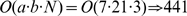 states and 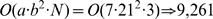 transitions suggested in “The composition algorithm”. Nonetheless, the extreme complexity of the ensemble model, despite the simplicity of the underlying model of RNA structure, makes clear the necessity for automated procedures for model construction. Dataset S1 gives the state space of the ensemble model constructed by the search algorithm and Dataset S2 the reduced model after eliminating windback states; both are in Graphviz format for visualization and show the state of the singlet transducer generating ancestral sequence as well as the states of the branch transducers generating observed sequences.

We implemented constrained maximum-likelihood inference of the structural alignment and ancestral structure of three extant sequences in a C++ program (Indiegram). For tractability, Indiegram uses the concept of fold envelopes described earlier to limit the fold space considered by the CYK algorithm, permitting structural information for the three extant sequences to be (optionally) supplied as input. If no structural information is supplied, then Indiegram uses a single-sequence SCFG to estimate a set of plausible folds [18], which are used to constrain the CYK algorithm.

The inference algorithms in Indiegram could be further constrained to enforce, for example, a fixed multiple alignment or a consensus structure for extant sequences. While experimentally-determined structures of individual RNAs are relatively rare, curated deep sequence alignments, such as those constructed for ribosomal RNAs [50], are frequently available for characterized RNA families. By constraining the inference algorithms with such sequence alignments, the memory and time complexity of the algorithms could be dramatically reduced. Such constraints can be naturally expressed with “alignment envelopes,” the alignment-space analogue of fold envelopes [18]. However, in this paper we focus on model construction and inference algorithms and postpone exploration of heuristics and constraints of these algorithms for future work.

### Reconstructing small RNAs with the TKFST model

While reconstructing large RNAs such as ribosomal subunits is currently computationally-inaccessible without further heuristics to constrain our algorithms, reconstructing small RNAs of biological interest will soon be feasible. Table 7 shows estimates of the memory and time required to reconstruct biologically-interesting subunits of the *nanos* 3′ translational control element and tRNAs, as well as two small RNAs which show significant structural divergence, the Y RNAs and Group II introns, and therefore promise to be interesting candidates for ancestral reconstruction. The reconstructed structures for three *nanos* 3′ translational control elements (TCEs) and three tRNAs, which could be analyzed given current computational limitations, can be found at http://biowiki.org/IndieGram; however, the phylogenetic trees relating the tested sequences have short branch lengths, making the reconstruction problem easy by forcing the reconstructed structures to be essentially-identical to those of one of the extant RNAs.

**Table 7:** Estimates of the memory and time required to reconstruct ancestral structures of three RNAs from several families of biological interest (as reported by Indiegram).

Family	Sequence lengths	Memory	Time
*nanos* 3′ TCE	61–64 nt	3 Gb	3 min
tRNA	69–73 nt	11 Gb	19 min
Y RNA	47–81 nt	33 Gb	70 min
Group II intron (domains V and VI)	76–91 nt	122 Gb	90 min

The *nanos* 3′ translational control element (TCE) sequences are the seed sequences of the corresponding RFAM family [5] and the three tRNA sequences are from the BRalibaseII database [55] (identifiers AB042432.1-14140_14072, Z82044.1-16031_16103 and AC008670.6-83725_83795). The group II intron sequences (identifiers Z00044.1-87253_87177, X57546.1-2817_2907 and X04465.1-2700_2775) are from BralibaseII [55]. The Y RNAs are hY1, hY4, and hY5 from [104]; sequence lengths exclude the conserved stem S1. The time estimates are for a 2.2 GHz AMD Opteron 848 CPU.

### Comparison of alignment methods

Guided by our experience with the *nanos* 3′ TCE and tRNA, where the reconstruction problem was made easy by the presence of a close outgroup, we conducted a simulation study of the dependence of reconstruction accuracy on outgroup branch length, with the further goal of comparing the performance of our reconstruction method (when simulating directly from the model) to simpler reconstruction methods that ignore either structure or phylogeny. (We here use the term “outgroup” loosely to denote the variable-length branch in our three-taxon study, where the other two branches are held at unit length.)

We simulated the evolution of RNAs under the TKFST model along three-taxon phylogenies (with one internal node), where we kept the branch lengths of two sibling species constant and varied the branch length of the outgroup between 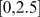 at steps of size 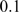. Parameters used in the simulation were 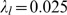 and 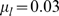 for loop sequence and 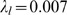 and 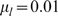 for stem sequence; the probability of a stem insertion was 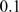. These yielded a mean loop length of 5 bp and a mean stem length of 2.33 bp, with 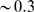 substructure indels per alignment. We selected alignments to reconstruct by requiring that there be at least two ancestral stems, loops of 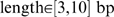 and stems of 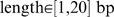; to reduce the complexity of our algorithms we additionally required that the sequences have 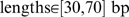.

We then attempted three-way multiple alignment (and, in some cases, reconstruction of the ancestor) using a variety of statistical inference algorithms. We sought insight as to the relative importance of the following factors in reconstructing ancestral RNA: (i) modeling the secondary structure; (ii) modeling the phylogenetic topology & branch lengths; (iii) using posterior-decoding algorithms to maximize the expected alignment *accuracy*, rather than picking the single *most likely* alignment [20],[51],[52].

The alignment programs we used in this benchmark were Indiegram (exact ML inference of alignment and ancestral structure, given phylogeny, descendant structures and correct model); Stemloc (a greedy ML heuristic, ignoring phylogeny in favor of a single-linkage clustering of the descendant structures); Stemloc-AMA (a posterior-decoding heuristic, maximizing the alignment's *expected accuracy* rather than its *likelihood*); and Handel (ML alignment under various indel models that ignore secondary structure completely). In detail, the reconstruction methods were

Stemloc : the Stemloc program was used to align the three sequences via single-linkage clustering with a Pair SCFG [18]. The structures of the leaf sequences were provided, but not the phylogenetic branch lengths. Instead of modeling a true phylogeny by introducing unobserved ancestral sequences, it just does single-linkage clustering of the observed sequences.

Stemloc-AMA : the Stemloc program was used to align the three sequences in “sequence annealing” mode, a posterior decoding method that attempts to optimize AMA, a sum-over-pairs alignment accuracy metric [20]. The structures of the leaf sequences were provided, but not the phylogenetic branch lengths. This program uses the same underlying pair SCFG as Stemloc, but instead of maximizing likelihood, it attempts to maximize an alignment accuracy metric.

TKF91: with the TKF91 model [44], the Handel package [30],[39],[40] was used to align the three extant sequences and reconstruct the ancestor. The correct phylogenetic tree and branch lengths were supplied (as they were for the Indiegram benchmark). The insertion, deletion and substitution rates for the TKF91 model were set equal to those of the loop submodel of TKFST. This may be understood as a naive sequence-only reconstruction that completely ignores basepair structure (i.e. the stem sub-model of TKFST).

Long Indel: with a single-event trajectory approximation to the long indel model [53], the Handel package was used to align the three extant sequences and reconstruct the ancestor. The correct phylogenetic tree and branch lengths were supplied. The deletion and substitution rates were set equal to those of the loop submodel of TKFST. The mean indel length was set equal to 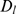, the mean number of bases that are created/removed by an insertion/deletion in the loop submodel of TKFST; the mean equilibrium sequence length (and thereby the insertion rate) was equal to 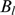, the mean number of bases in TKFST at equilibrium (“A simple model of RNA structural evolution” has formulae for these quantities in terms of the TKFST rate parameters). This model improves on the previous model (TKF91) by introducing affine gap penalties.

We measured alignment accuracy, under the simplifying assumption that this correlates well with ancestral reconstruction accuracy.

**Figure 10:**
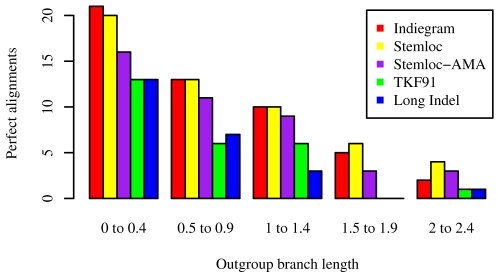
Dependence of perfect alignment rate on alignment method and outgroup branch length. Perfect alignment is when a given alignment program estimates the alignment 100% correctly, with no errors. Simulating the evolution of three structural RNAs under the TKFST model, we investigated the dependency of perfect alignment rate on outgroup branch length. The simulation included two sister species at unit distance from the ancestral sequence, plus one “outgroup” whose branch length 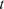 was varied between 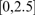 by selecting 25 equally spaced values of 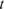 in this range, spaced 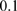 apart. (A unit-length branch here corresponds to one expected substitution per site in loop sequence.) We simulated 25 alignments for each value of 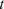, using TKFST model parameters described in the text. Since the perfect alignment rate is rather low, we further aggregated the 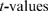 into bins of five; thus, for example, the bin named “
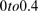
” includes 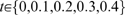 and represents 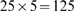 trials in total. The perfect alignment rate was measured for various statistical alignment inference procedures. These procedures are described in the text, but may be summarized very briefly as ML under the true model (“Indiegram”); greedy approximate-ML progressive alignment by single-linkage clustering with pair SCFGs (“Stemloc”); sequence annealing, a form of posterior decoding to maximize a sum-over-pairs accuracy metric, using pair SCFGs to get the posterior probabilities (“Stemloc-AMA”); statistical alignment using the TKF91 model, i.e. linear gap-penalties (“TKF91”); and statistical alignment using a long-indel model, i.e. affine gap-penalties (“Long Indel”).

We first consider the *perfect alignment rate*; that is, the number of times each method gets the alignment exactly correct. Theory predicts that Maximum Likelihood inference, using the correct model and parameters, should be asymptotically optimal (if one only counts perfect guesses). Inspecting Figure 10, we find this to be almost the case; the exception is when the outgroup is very distant and the bins may be undersampled (the departure from prediction that we observe for low-identity alignments is not statistically significant: when the optimal success rate drops below 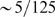, then 125 trials are probably insufficient to compare two near-optimal methods). We also note that the ML version of Stemloc is near-optimal, despite the Stemloc pair-SCFG being slightly different from the TKFST pair-SCFG in parameterization and structure (e.g. Stemloc 's grammar does not allow insertion/deletion of entire substructures). The ML version of Stemloc is also observed to have a slightly higher perfect alignment rate than the posterior-decoding version (Stemloc -AMA). Finally, we note that the structure-blind models (TKF91 and Long Indel) perform consistently worse than the structure-aware methods; furthermore, both linear (TKF91) and affine (Long Indel) gap-penalties perform equally bad in this test (note that the TKFST model, from which the true alignments were simulated, does not allow long-indel events, which may partly explain why affine gap-penalties do not help in this benchmark).

**Figure 11:**
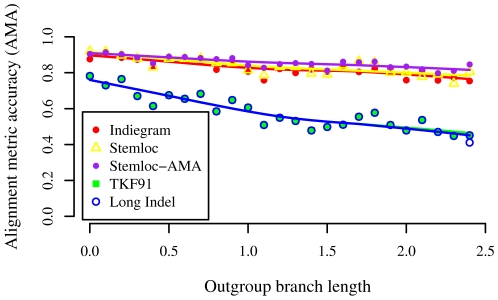
Dependence of alignment metric accuracy on alignment method and outgroup branch length. We simulated the evolution of three structural RNAs under the TKFST model. The simulation included two sister species at unit distance from the ancestral sequence, plus one “outgroup” whose branch length 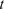 was varied between 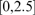 by selecting 25 equally spaced values of 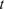 in this range, spaced 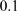 apart. We then simulated 25 alignments for each value of 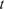, using TKFST model parameters described in the text. The Alignment Metric Accuracy (AMA) is, roughly, the proportion of residues that are correctly aligned, averaged over all pairs of sequences (see [54] for a precise definition; we set the AMA Gap Factor to 1). The AMA between the true alignment and the inferred alignment was measured for various statistical alignment inference procedures. These procedures are described in the text, but may be summarized very briefly as ML under the true model (“Indiegram”); greedy approximate-ML progressive alignment by single-linkage clustering with pair SCFGs (“Stemloc”); sequence annealing, a form of posterior decoding to maximize a sum-over-pairs accuracy metric, using pair SCFGs to get the posterior probabilities (“Stemloc-AMA”); statistical alignment using the TKF91 model, i.e. linear gap-penalties (“TKF91”); and statistical alignment using a long-indel model, i.e. affine gap-penalties (“Long Indel”).

A subtly different ranking emerges from consideration of the *alignment accuracy*. In Figure 11, we abandon the all-or-nothing metric of counting only perfect alignments, instead using a metric that shows what proportion of the alignment is correct. Specifically, we plot the *Alignment Metric Accuracy* (AMA) as a function of outgroup branch length. AMA measures the proportion of residues which are correctly aligned, averaged over all pairs of sequences [54]. Figure 4 reveals that Stemloc-AMA (which attempts to find the alignment with the maximum expected AMA) edges out both Indiegram and the ML version of Stemloc (both of which attempt to find the alignment with the maximum likelihood). These results, compared to the subtly different story told by the perfect alignment rate, underscore the point that benchmark results for alignment methods can depend exquisitely on the choice of accuracy metric. The superiority of ML methods is only assured in terms of perfect alignment rate, and not necessarily other accuracy metrics.

Taken together, these results suggest that the most important factor distinguishing the various models we have examined is the incorporation of some form of basepair structure: structure-blind Handel (regardless of linear *vs* affine gap penalty) performs much worse than the structure-aware SCFG methods. Intuitively, this is to be expected: whenever a basepair-aware method aligns one half of a basepair, it gets the other nucleotide correctly aligned for free. In benchmarks of RNA multiple alignment programs, structure-aware scoring schemes routinely outperform structure-blind scoring schemes [55],[56]. Since we know that modeling structure is very important, it's not too surprising that it turns out to be the most important of the factors we considered.

The second most important, amongst the factors we have considered in this experiment, is selection of the most appropriate objective function for the task at hand (c.f. perfect alignment rate *vs* AMA), followed by use of the correct posterior-decoding algorithm for the chosen objective function (c.f. Stemloc *vs* Stemloc-AMA). This is a subtle but important point: before deciding exactly what inference algorithm we're going to use to reconstruct ancestral sequences, we need to decide whether we want to maximize (a) the probability that our reconstructed sequence is 100% correct, (b) the expected number of nucleotides that are correctly reconstructed, (c) the expected number of base-pairs that are correctly reconstructed, (d) the expected number of stems that are correctly reconstructed, (e) some other metric. Each of these metrics would require a slightly different inference algorithm.

Lastly, the fine details of the scoring scheme—including branch lengths, substitution scores, gap penalties and so forth—appear to be the least important of the factors we considered, yielding observable differences only when all other aspects of the inference procedure were more-or-less equal. While such details of the model may affect reconstruction quality, they appear to have very minor influence on alignment quality.

## Discussion

Following the conception of paleogenetics [57], a large number of synthetic reconstructions of ancestral protein sequences have been reported in the literature [58]–[65]. There is also scientific interest in reconstructing DNA sequences [33], [66]–[71]. Given the importance of the RNA world hypothesis to current discussions of the origin of life [72]–[78], the many modern-day relics of this world [79]–[82] and the recent proposal of a structural model for the primordial ribosome [2], we believe that phylogenetic reconstruction of ancient RNA is a significant problem, deserving of strong bioinformatics support.

The work reported in this paper builds on extensive prior art in the areas of evolutionary modeling and ancestral reconstruction. Reviewing all of this would take several books, but we can note some key references. The reconstruction of ancient sequences was first proposed in 1963 by Pauling and Zuckerkandl [57]; current applications of this idea, mostly using substitution models, are surveyed in the book edited by Liberles [83]. Many algorithms in phylogenetics implicitly reconstruct substitution histories, whether by parsimony [84],[85] or likelihood [22]. There is a substantial body of work to model indels on phylogenies [30], [35], [39], [44], [53], [86]–[92]. Recent work has extended these ideas to the reconstruction of indel histories [93],[94], particularly at the genomic scale [33],[95]. There is also prior work in computational linguistics on the theory of transducers for sequences [96] and parse trees [36],[37],[97],[98] (from which we take the terms “string transducer” and “parse-tree transducer”). We draw on the bioinformatics literature for SCFGs [10],[11],[99], especially Pair SCFGs [14],[18],[19] and phylogenetic SCFGs [16]. In particular, an early example of a pairwise conditional model 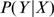 for structure-dependent RNA evolution was given by Eddy *et al* [12]. A conditional framework similar to ours in some respects is described by Sakakibara *et al* [100]. The dynamic programming inference algorithms for multiple-sequence SCFGs are closely related to the protosequence algorithm of Sankoff [42].

While we have focused on the TKF Structure Tree model in our Results, our model-construction algorithm is applicable to any model of the evolution of secondary structure which can be expressed as a Pair SCFG. Realistic structural and thermodynamic effects—such as base-stacking or loop length distributions—can, in principle, be incorporated. Other phenomena of RNA evolution may prove more difficult: modeling helix slippage with a branch transducer is awkward, let alone more radical changes in structure; pseudoknots, too, are impossible with the models we have described here. Even so, variants of our models could be used for proposing candidate alignments for more accurate scoring by such models.

An implementation of inference algorithms for models on the three-taxon phylogeny is sufficient to construct a MCMC sampling algorithm over many sequences on an arbitrary phylogeny. A sketch of such a sampling algorithm is as follows: at each step of the sampling algorithm, we re-sample the sequence and structure of the ancestral node 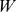, conditioned on the sequences and structures of 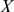, 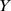 and 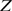. The structural alignment of all four sequences can change at each step, providing for fast mixing and guaranteeing ergodicity. This move is similar to the sampler proposed by [31] for models with a HMM structure. Note that this, in principle, permits construction of a crude sampler to simultaneously infer phylogeny as well, by proposing and accepting or rejecting changes to the underlying tree as well as the implied structural alignment.

Reconstructing structural changes of large RNAs using the three-way sampling kernel which we have described would require resources far in excess of those currently available; barring the availability of supercomputers with terabytes of memory, such algorithms will only be feasible for short RNAs (Table 7). A promising direction is to consider variations on the three-way sampling kernel, such as the importance-sampling approach described for the TKF model by [32]. This approach first proposes an ancestor 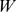 by aligning extant sequence 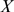 to 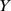 (ignoring 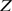); then, in a second step, the proposed 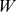 is independently aligned to 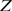. The proposed three-way alignment and reconstruction is then randomly accepted (or rejected) using a Hastings ratio based on the three-way transducer composition. The complexity of this kernel is the same as the pairwise case; with suitable constraints, this is feasible for RNA grammars on present hardware, at least for ribosomal domains (if not yet whole subunits—although pairwise alignment of those should also be possible soon). The approach of Redelings and Suchard therefore merits future consideration in the context of modeling the evolution of RNAs on a tree.

An alternative MCMC scheme for sampling RNA phylogeny, structure and alignment was developed for the SimulFold program [101]. SimulFold does not use a strictly normalized probabilistic model, resulting in some oddities in the ways that structure and indels interact (for example, it does not penalize deletion of one half of a basepair). Currently, it is not clear how appropriate SimulFold would be for ancestral reconstruction, although it has several advantages (e.g., explicit treatment of pseudoknots). Of course, MCMC kernels are inherently adaptable to other purposes: the MCMC moves developed for SimulFold may be useful for inference under different models.

This paper focuses on the case where the tree topology is known, but many of the methods which we have described can be extended to the more general case where none of the possible constraints (phylogeny, structure or alignment) are final. For example, the probabilistic framework readily allows us to compare likelihoods of two different phylogenetic trees by constructing a composite transducer for each tree. Thus, the MCMC samplers described above for alignments could, in principal, be extended to phylogenies (albeit at a computational cost).

While MCMC provides the most information about the posterior distribution of evolutionary histories, in practice a maximum likelihood inference may be adequate (and typically much faster). The progressive profiling used by the Ortheus program for reconstructing ancestral genomes is promising [33]. This approach is similar to a progressive multiple alignment algorithm, in that it proceeds via a single postorder (leaf-to-root) traversal of the phylogeny. As each node is visited, a profile is generated for that node, by aligning the profiles of its children to a composite transducer using DP, then sampling a finite number of traceback paths through the DP matrix. The profile is not linear: the sampled paths instead form a reticulate network, a.k.a. a partial order graph [102]. An equivalent of Ortheus for RNA reconstruction should be possible, representing the intermediate profiles using transducers.

Given the excellent performance of Stemloc-AMA 's sequence annealing, particularly when measured using its own scoring metric (AMA), such posterior-decoding methods should also be considered for reconstruction.

In summary, the evolutionary models and algorithms we have described form a systematic theoretical platform on which we can test different optimization and sampling strategies for studying the structural evolution of RNA gene families in detail. Stochastic grammars are powerful tools for this task, although they will not be the only tools we need, particularly as we move towards modeling RNA evolution in greater detail. Our hope is that these algorithms will allow us to test and refine our understanding of RNA evolution by computational reconstruction and (eventually) direct experimental investigation of early ribonucleic machines.

## Supporting Information

Text S1 Formal description of transducer composition algorithm yielding phylogenetic grammar rules
(0.15 MB PDF)

Text S2 Algorithm for elimination of null cycles and empty bifurcations from general SCFGs
(0.13 MB PDF)

Text S3 Description of software tools
(0.09 MB PDF)

Text S4 Example presentation of TKFST inference grammars in Indiegram format
(0.14 MB PDF)

Dataset S1 Grammar for three-taxon TKFST model returned by transducer composition algorithm (graphviz dot format)
(0.03 MB TXT)

Dataset S2 Grammar for three-taxon TKFST model returned by transducer composition algorithm, after eliminating windback states (graphviz dot format)
(0.06 MB TXT)
