# Supplementary material for: Evolutionary Triplet Models of Structured RNA
Source: PLoS Comput Biol. 2009 Aug 28;5(8):e1000483. doi: 10.1371/journal.pcbi.1000483 (PMC2725318; doi:10.1371/journal.pcbi.1000483)
Supplement: Text S1 — Formal description of transducer composition algorithm yielding phylogenetic grammar rules (0.15 MB PDF) [file pcbi.1000483.s001.pdf]

Evolutionary triplet models of structured RNA: Text S1

Robert K. Bradley[1], Ian Holmes[1,2,*]

1 Biophysics Graduate Group, University of California, Berkeley, CA, USA

2 Department of Bioengineering, University of California, Berkeley, CA, USA

* E-mail: indiegram@postbox.biowiki.org

Contents

1 Two-sequence transducer models **3**

1.1 Formal grammars 3

1.2 States, transitions and emissions 3

2 Multiple-sequence transducer models **6**

2.1 The guide tree 6

2.2 States, transitions and emissions 6

2.3 Formal grammars 8

2.4 Constructing the state graph 8

2.5 Allowed transitions 9

1 Two-sequence transducer models

We here give formal definitions of two-sequence models and the state machines which generate the factored probability distribution $P(X, Y|\Delta T) = P(X) \cdot P(Y|X, \Delta T)$, where the marginal $P(X)$ is generated by a **singlet transducer** and the conditional $P(Y|X, \Delta T)$ by a **branch transducer**. We refer to the branch transducer as θ, so the conditional distribution is more precisely $P(Y|X, \Delta T, \theta)$.

1.1 Formal grammars

There is a close relationship between formal grammars and the singlet and branch transducer abstract state machines. By labeling the nonterminals of a regular or stochastic context-free grammar (SCFG) as states of an abstract machine, allowing these states to absorb and emit appropriate terminal symbols, and carefully assigning transition and emission weights, we can create a state machine which generates the same language, with the same distribution of weights, as that produced by the original grammar.[1] We therefore use the terms "nonterminal" and "state" interchangeably and refer to the "parse tree" generated by singlet and branch transducers.

Phrased more precisely, there is an isomorphism between the singlet and branch transducers of two-sequence models and Pair SCFGs. Practically speaking, this means that for every joint distribution $P(X, Y|\Delta T)$ generated by a singlet and branch transducers, there exists a Pair SCFG which generates the same distribution.

1.2 States, transitions and emissions

A singlet transducer has states of type `Start` and `Insert`. Each state $\phi \in \Phi$ of the branch transducer has type

$$\text{type}(\phi) \in \{\texttt{Start}, \texttt{Insert}, \texttt{Match}, \texttt{Wait}, \texttt{End}\}\,.$$

State typing is as follows:

- A `Start` state begins a branch of the parse tree.
- An `Insert` state emits, but does not absorb, symbols.
- A `Match` state absorbs and (possibly) emits symbols.

[1] We speak of weights associated with a transition rather than probabilities in order to allow for more general models.

- A `Wait` state is a null state which allows a branch transducer to "pause" while it waits for an input symbol from another transducer. In the two-sequence case, the input symbols is emitted by the singlet transducer generating the ancestral sequence.
- An `End` state ends a branch of the parse tree.

The names of state types are similar to the {`Start`, `Insert`, `Match`, `End`} states of the familiar Pair HMM. Deletions are handled as a special case by states of type `Match` which absorb but do not emit symbols pairs. Only states of type `Match` can absorb symbol pairs (x, y).

Bifurcations in the grammar, when considered as emission of nonterminals, can be handled analogously to terminal emission. States $\phi : \text{type}(\phi) \in \{$`Insert`, `Match`$\}$ can emit nonterminal pairs $(c\,d)$, where c or d can be the null symbol, and the emitted pairs $(c\,d)$ can be absorbed by states of type `Match`.

The emission weight of a nonterminal pair $(c\,d)$ from the state $b : \text{type}(b) =$ `Match`, conditional on absorption of a nonterminal pair $(l\,m)$, is

$$e_b(c\,d|l\,m, \theta)\,.$$

The functions emit() and absorb() are defined to return the emission or absorption of a particular state,

$$\text{emit}(\phi) := \begin{cases} (x, y) & x, y = \text{terminal or null} \\ (c\,d) & c, d = \text{nonterminal or null.} \end{cases}$$

$$\text{absorb}(\phi) := \begin{cases} (x, y) & x, y = \text{terminal or null} \\ (c\,d) & c, d = \text{nonterminal or null.} \end{cases}$$

We frequently use the notation ${}^{uv}\phi^{xy}$ to indicate that a state of type `Match` absorbs a symbol pair (u, v) and emits a pair (x, y). The notation for bifurcations is slightly different: A bifurcation state which left-emits a nonterminal d and makes a transition to a state ϕ with weight 1 is written as $\mathtt{B}[\mathtt{d}\,\phi]$.

A transition between states a and b of the branch transducer θ has a weight

$$t(a, b|\theta) = t(a \rightarrow b|\theta).$$

Terminal emission is handled by states $\phi : \mathrm{type}(\phi) \in \{\texttt{Match}, \texttt{Insert}\}$, which emit symbol pairs (x, y), where x or y can be the null symbol. In this paired-emission perspective, left single-terminal emissions x are represented as $(x\,\mathrm{null})$; right single-terminal emissions are handled similarly. The weight of an emission of a terminal pair (x, y) from a state $b : \mathrm{type}(b) = \texttt{Match}$, conditioned on absorption of a terminal pair (u, v), is

$$e_b(x, y|u, v, \theta)\,.$$

Recall that the emission weights of states of type `Match` are defined conditioned upon the absorbed symbols.

2 Multiple-sequence transducer models

We can use our two-sequence models to construct a model of many sequence related by a guide phylogenetic tree. The guide tree specifies the (conjectured) phylogenetic relationship of all sequences. A singlet transducer, which emits, but does not absorb, symbols, lies at the root of the guide tree and serves as a generative model of the ancestral sequence. A branch transducer represents the evolution of an ancestral sequence into a single descendant sequence, that is, the action of evolution along the single-branch tree (Ancestor → Descendant). To represent the evolution of an ancestral sequence into many descendant sequences (whose phylogenetic relationship is specified by the guide tree), we place a branch transducer on each branch of the guide tree to create a multiple-sequence model.

If the branch grammar has no bifurcations and only left or right emissions are allowed, then the language generated is a regular string language and the corresponding jointly normalized abstract state machine is an HMM. In this simplest case our formalism for creating a multiple-sequence model reduces to that given by [1] for combining HMMs on a guide tree.

2.1 The guide tree

The nodes of the tree are labeled $1, \ldots, N$ in the order reached by any preorder depth-first traversal of the tree. The length of each branch (parent$(m) \rightarrow m$) is given by the evolutionary time t_m. To specify ancestor-descendant relationships, we introduce notation: $m \rhd n$ ($m \ntriangleright n$) means node m is descended from (not descended from) node n, and $m \trianglerighteq n$ ($m \ntrianglerighteq n$) means node m is descended from or identical to (not descended from and not identical to) node n.

2.2 States, transitions and emissions

The multiple-sequence model is formed by the composition/intersection of $(N-1)$ branch transducers such that there is a branch transducer on each branch (parent$(m) \rightarrow m$); $m = 2, \ldots, N$ of the guide tree and a singlet transducer at the root node. Our framework allows for the placement of different branch transducers, with a unique set of nonterminals (state space) Φ and allowed transitions between states and corresponding weights, on each branch. $\theta^{(m)}$ denotes the branch transducer governing evolution along the branch (parent$(m) \rightarrow m$) of the guide tree.

States of the multiple-sequence model are represented by as N-dimensional vectors $\boldsymbol{a}$,

$$\boldsymbol{a} = \begin{pmatrix} a_1 \\ \vdots \\ a_N \end{pmatrix} .$$

These states are typed as

$$\text{type}(\boldsymbol{a}) \in \{\texttt{Start}, \texttt{Emit}, \texttt{Bifurcation}, \texttt{Null}, \texttt{End}\} .$$

State typing is as follows:

- A `Start` state begins a branch of the parse tree of one or more of the N sequences on the phylogenetic tree.
- An `Emit` state emits symbols (terminals) to one or more of the N sequences.
- A `Bifurcation` state corresponds to a bifurcation in the parse tree of one or more of the N sequences.
- A `Null` state corresponds to any non-`End` state which represents neither an emission or bifurcation.
- An `End` state ends a branch of the parse tree.

States are typed according to the transition by which they are reachable (details of the typing are given in Section 2.5).

Transitions and emissions of the multiple-sequence model are defined in terms of the transitions and emissions of the branch transducers at each node as well as the singlet transducer at the root node. The weight of a transition $\boldsymbol{a} \to \boldsymbol{b}$ is therefore

$$t(\boldsymbol{a}, \boldsymbol{b}) = \prod_{m | a_m \neq b_m} t\left(a_m, b_m | \theta^{(m)}\right) , \tag{1}$$

and the weight of an emission $(\boldsymbol{x}\,\boldsymbol{y})$ from a state $\boldsymbol{b}$ is

$$_{\boldsymbol{b}}(\boldsymbol{x}\,\boldsymbol{y}) = \prod_m e_{b_m}\left(x_m\, y_m | x_{\text{parent}(m)}\, y_{\text{parent}(m)}, \theta^{(m)}\right) . \tag{2}$$

We frequently will not explicitly write out the conditional dependence on the absorbed terminals $(x_{\mathrm{parent}(m)}\, y_{\mathrm{parent}(m)})$, but the reader should keep in mind that in general emission weights will depend on the absorbed symbols.

2.3 Formal grammars

Analogously to the case with two-sequence models (Section 1.1), there exists a one-to-one mapping between the multiple-sequence models generated by our model-construction algorithm and multi-sequence SCFGs. In other words, given a singlet and branch transducers of a two-sequence model, as well as a guide tree relating the extant sequences, there exists a corresponding multi-sequence SCFG which generates the same joint probability distribution $P(X_1, ..., X_n)$.

2.4 Constructing the state graph

As described in the paper, we need a way to efficiently construct the state graph of the multi-sequence model, where the state graph consists of a list of accessible states and the possible transitions between them. This state graph can be constructed by an uninformed depth-first search, where at each step of the search we obtain the possible child nodes by applying one of the following possible transitions of the multi-sequence model:

1. **Null transition**: The state of a single branch transducer is updated, with no terminal emission or bifurcation.

2. **Terminal emission**: A state makes a transition to a `Insert` state. The emitted symbol is passed down the guide tree to all descendant branch transducers, which transition to states of type `Match`.

3. **Bifurcation**: A state makes a transition to a special `Insert` state which emits a new branch of the parse tree. The emitted symbol is passed down the guide tree to all descendant branch transducers, which transition to states of type `Match`.

4. **End transition**: The singlet transducer associated with the root sequence can transition to the `End` state, signaling that this parse tree is finished.

Each of these transitions is explained in detail in the following section.

2.5 Allowed transitions

Following [1], we let transitions of the multi-sequence model begin at the active node and cascade down the guide tree as appropriate. Unless defined otherwise, node n is the active node of the multi-sequence model with state $\boldsymbol{a}$,

$$n = n(\boldsymbol{a}) \tag{3}$$

$$= \mathrm{argmax}_m \left\{ \mathrm{type}(a_m) \notin \{\texttt{Wait}, \texttt{End}\} \right\} . \tag{4}$$

Each possible allowed transition is obtained by making a valid change (updating) the state of the singlet or one or more of the branch transducers of the multi-sequence model.

Null Transitions

$$\begin{pmatrix} a_1 \\ \vdots \\ a_N \end{pmatrix} \to \begin{pmatrix} b_1 \\ \vdots \\ b_N \end{pmatrix}$$

$$\mathrm{type}(\boldsymbol{b}) = \texttt{Null} \tag{5}$$

$$\mathrm{Weight}(\boldsymbol{a} \to \boldsymbol{b}) = t(\boldsymbol{a}, \boldsymbol{b}) \tag{6}$$

$$= t\left(a_n, b_n | \theta^{(n)}\right) . \tag{7}$$

This transition updates the state of the branch transducer at the active node n of the guide tree, leaving the rest unchanged, with no corresponding terminal emission or bifurcation in the grammar. Nodes other than the active node do not change state, $a_m = b_m \, \forall \, m \neq n$, and the only allowable transitions of this form are to states $b_n : \mathrm{type}(b_n) \in \{\texttt{Start}, \texttt{Wait}\}$. Transitions to states of type `Insert` or `Match` result in emissions, and transitions to the end state `End` are handled as a special case.

Terminal Emission

$$\begin{pmatrix} a_1 \\ \vdots \\ a_N \end{pmatrix} \to \begin{pmatrix} x_1 \\ \vdots \\ x_N \end{pmatrix} \begin{pmatrix} b_1 \\ \vdots \\ b_N \end{pmatrix} \begin{pmatrix} y_1 \\ \vdots \\ y_N \end{pmatrix}$$

$$\text{type}(\boldsymbol{b}) = \texttt{Emit} \tag{8}$$

$$\text{Weight}(\boldsymbol{a} \to \boldsymbol{x}\,\boldsymbol{b}\,\boldsymbol{y}) = t(\boldsymbol{a}, \boldsymbol{b}) \cdot {}_{\boldsymbol{b}}(\boldsymbol{x}\,\boldsymbol{y}) \tag{9}$$

$$= \left[\prod_{m|a_m \neq b_m} t\left(a_m, b_m|\theta^{(m)}\right) \right] \cdot \left[\prod_m e_{b_m}\left(x_m\, y_m|\theta^{(m)}\right) \right], \tag{10}$$

where we are defining states with no emissions to emit (null null) with weight 1, $e_{b_m}\left(x_m\, y_m|\theta^{(m)}\right) = 1$ if $(x_m\, y_m) = $ (null null) and emit$(b_m) =$ null.

The active node n makes a transition to a state $b_n :$ type$(b_n) = \texttt{Insert}$, emitting a terminal symbol pair emit$(b_n) = (x_n\, y_n)$. This symbol pair is passed down the guide tree to descendant nodes $\{m|m \rhd n, \text{type}(a_m) \neq \texttt{End}\}$, forcing them to make a transition from states of type `Wait` to states of type `Match`, which can absorb terminal pairs (x, y). Qualitatively, this transition and emission could represent the evolution of two paired nucleotides along the subtree rooted at node n of the complete guide tree. If one of $\boldsymbol{x}$ or $\boldsymbol{y}$ is null, then (8) could represent the evolution of a single unpaired nucleotide along the subtree rooted at node n.

Left emission. All terminals $\boldsymbol{y}$ in the transition $\boldsymbol{a} \to \boldsymbol{x}\,\boldsymbol{b}\,\boldsymbol{y}$ (8) are null.

Nodes $m \not\trianglerighteq n$: $a_m = b_m$.

$(x_m\, y_m) =$ (GAP null).

Node n: $b_n :$ type$(b_n) = \texttt{Insert}$, $\exists$ a transition $a_n \to b_n$ in the branch transducer $\theta^{(n)}$.

$(x_n\, y_n) =$ emit(b_n).

Nodes $m \rhd n$: Either $a_m = b_m$, emit$(b_{\text{parent}(m)}) =$ null or type$(a_m) = \texttt{End}$

or emit$(b_{\text{parent}(m)}) =$ absorb$(b_m) = (x_{\text{parent}(m)}$ null).

$$(x_m\, y_m) = \begin{cases} (\text{GAP null}) & \text{emit}(b_m) = \text{null} \\ \text{emit}(b_m) & \text{emit}(b_m) \neq \text{null} \end{cases}$$

Right emission. All terminals $\boldsymbol{x}$ in the transition $\boldsymbol{a} \to \boldsymbol{x}\,\boldsymbol{b}\,\boldsymbol{y}$ (8) are null.

Nodes $m \ntrianglerighteq n$: $a_m = b_m$.

$(x_m\, y_m) = (\text{null}\,\text{GAP})$.

Node n: $b_n : \text{type}(b_n) = \texttt{Insert}$, $\exists$ a transition $a_n \to b_n$ in the branch transducer $\theta^{(n)}$.

$(x_n\, y_n) = \text{emit}(b_n)$.

Nodes $m \triangleright n$: Either $a_m = b_m$, $\text{emit}(b_{\text{parent}(m)}) = \text{null}$ or $\text{type}(a_m) = \texttt{End}$

or $\text{emit}(b_{\text{parent}(m)}) = \text{absorb}(b_m) = (\text{null}\, y_{\text{parent}(m)})$.

$$(x_m\, y_m) = \begin{cases} (\text{null}\,\text{GAP}) & \text{emit}(b_m) = \text{null} \\ \text{emit}(b_m) & \text{emit}(b_m) \neq \text{null}. \end{cases}$$

Paired emission. There is at least one non-null terminal in both $\boldsymbol{x}$ and $\boldsymbol{y}$ in the transition $\boldsymbol{a} \to \boldsymbol{x}\,\boldsymbol{b}\,\boldsymbol{y}$ (8).

Nodes $m \ntrianglerighteq n$: $a_m = b_m$.

$(x_m\, y_m) = (\text{GAP}\,\text{GAP})$.

Node n: $b_n : \text{type}(b_n) = \texttt{Insert}$, $\exists$ a transition $a_n \to b_n$ in the branch transducer $\theta^{(n)}$.

$(x_n\, y_n) = \text{emit}(b_n)$.

Nodes $m \triangleright n$: Either $a_m = b_m$, $\text{emit}(b_{\text{parent}(m)}) = \text{null}$ or $\text{type}(a_m) = \texttt{End}$

or $\text{emit}(b_{\text{parent}(m)}) = \text{absorb}(b_m) = (x_{\text{parent}(m)}\, y_{\text{parent}(m)})$.

$$(x_m\, y_m) = \begin{cases} (\text{GAP}\,\text{GAP}) & \text{emit}(b_m) = \text{null} \\ \text{emit}(b_m) & \text{emit}(b_m) \neq \text{null}. \end{cases}$$

Bifurcations

$$\begin{pmatrix} a_1 \\ \vdots \\ a_N \end{pmatrix} \to \begin{pmatrix} c_1 \\ \vdots \\ c_N \end{pmatrix} \begin{pmatrix} b_1 \\ \vdots \\ b_N \end{pmatrix} \begin{pmatrix} d_1 \\ \vdots \\ d_N \end{pmatrix}$$

$$\text{type}(\boldsymbol{b}) = \texttt{Bifurcation} \tag{11}$$

$$\text{Weight}(\boldsymbol{a} \to \boldsymbol{c}\,\boldsymbol{b}\,\boldsymbol{d}) = t(\boldsymbol{a}, \boldsymbol{b}) \cdot {}_{\boldsymbol{b}}(\boldsymbol{c}\,\boldsymbol{d}) \tag{12}$$

$$= \left[\prod_{m|\, a_m \neq b_m} t\left(a_m, b_m | \theta^{(m)}\right) \right] \cdot \left[\prod_m e_{b_m}\left(c_m\, d_m | \theta^{(m)}\right) \right]. \tag{13}$$

where we are defining the emission weight of the End nonterminal to be 1, $e_{b_m}\left(c_m\, d_m|\theta^{(m)}\right) = 1$ if $c_m = \texttt{End}$ or $d_m = \texttt{End}$.

Bifurcations are handled similarly to terminal emission. The active node n can undergo a bifurcation by making a transition $a_n \to b_n$, b_n : type$(b_n) = \texttt{Insert}$, emitting a pair of nonterminals emit$(b_n) = (c_n\, d_n)$. Descendant nodes $\{m|m \rhd n, \text{type}(a_m) \neq \texttt{End}\}$ are forced to make a transition from states of type Wait to states of type Match which can absorb nonterminal pairs $(c\, d)$. All emissions are pairwise, so left and right bifurcations are represented as pairs $(\boldsymbol{c}\,\boldsymbol{d})$ where either $\boldsymbol{c}$ or $\boldsymbol{d}$ is null. If $\boldsymbol{d}$ is null, then (11) could represent the insertion and subsequent evolution of a new RNA stem-loop structure.

Left bifurcation. All nonterminals $\boldsymbol{d}$ in the transition $\boldsymbol{a} \to \boldsymbol{c}\,\boldsymbol{b}\,\boldsymbol{d}$ (11) are null. The nonterminals $\boldsymbol{c}$ are the "new" states (for example, corresponding to a newly formed stem); the nonterminals $\boldsymbol{b}$ are the states which will generate the (evolved) ancestral sequence.

Nodes $m \not\trianglerighteq n$: $a_m = b_m$.

$(c_m\, d_m) = (\texttt{End}\,\text{null})$.

Node n: b_n : type$(b_n) = \texttt{Insert}$, $\exists$ a transition $a_n \to b_n$ in the branch transducer $\theta^{(n)}$.

$(c_n\, d_n) = \text{emit}(b_n)$.

Nodes $m \rhd n$: Either $a_m = b_m$, emit$(b_{\text{parent}(m)}) = \text{null}$ or type$(a_m) = \texttt{End}$

or emit$(b_{\text{parent}(m)}) = \text{absorb}(b_m)$

$(c_m\, d_m) = \text{emit}(b_m)$.

Right bifurcation. All nonterminals $\boldsymbol{c}$ in the transition $\boldsymbol{a} \to \boldsymbol{c}\,\boldsymbol{b}\,\boldsymbol{d}$ (11) are null. The nonterminals $\boldsymbol{d}$ are the "new" states; the nonterminals $\boldsymbol{b}$ are the states which will generate the (evolved) ancestral sequence.

Nodes $m \not\trianglerighteq n$: $a_m = b_m$.

$(c_m\, d_m) = (\text{null}\,\texttt{End})$.

Node n: b_n : type$(b_n) = \texttt{Insert}$, $\exists$ a transition $a_n \to b_n$ in the branch transducer $\theta^{(n)}$.

$(c_n\, d_n) = \text{emit}(b_n)$.

Nodes $m \rhd n$: Either $a_m = b_m$, emit$(b_{\text{parent}(m)}) = \text{null}$ or type$(a_m) = \texttt{End}$

or emit$(b_{\text{parent}(m)}) = \text{absorb}(b_m)$

$(c_m\, d_m) = \text{emit}(b_m)$.

Paired bifurcation There is at least one non-null nonterminal in both $\boldsymbol{c}$ and $\boldsymbol{d}$ in the transition $\boldsymbol{a} \to \boldsymbol{c}\,\boldsymbol{b}\,\boldsymbol{d}$ (11). The nonterminals $\boldsymbol{c}$ and $\boldsymbol{d}$ are both "new" states; the nonterminals $\boldsymbol{b}$ are the states which will generate the (evolved) ancestral sequence.

Nodes $m \ntrianglerighteq n$: $a_m = b_m$.

$(c_m\, d_m) = (\texttt{End}\,\texttt{End})$.

Node n: $b_n : \text{type}(b_n) = \texttt{Insert}$, $\exists$ a transition $a_n \to b_n$ in the branch transducer $\theta^{(n)}$.

$(c_n\, d_n) = \text{emit}(b_n)$.

Nodes $m \triangleright n$: Either $a_m = b_m$, $\text{emit}(b_{\text{parent}(m)}) = \text{null}$ or $\text{type}(a_m) = \texttt{End}$

or $\text{emit}(b_{\text{parent}(m)}) = \text{absorb}(b_m)$

$(c_m\, d_m) = \text{emit}(b_m)$.

This paired-bifurcation is included for completeness–for example, it could be used to model symmetric loops–but it increases the complexity of grammar parsing.

Transition to `End`

$$\begin{pmatrix} a_1 \\ \vdots \\ a_N \end{pmatrix} \to \texttt{End}$$

$$\text{Weight}(\boldsymbol{a} \to \texttt{End}) = t(\boldsymbol{a}, \texttt{End}) \tag{14}$$

$$= \prod_{m \mid \text{type}(a_m) \neq \texttt{End}} t\left(a_m, \texttt{End} | \theta^{(m)}\right). \tag{15}$$

The singlet transducer associated with the highest active ancestral node,

$$\hat{n} = \text{argmin}_m \left\{ \text{type}(a_m) \in \{\texttt{Start},\ \texttt{Insert}\} \right\} \tag{16}$$

can make a transition to the state `End`, forcing the entire multi-sequence model to transition to `End`.[2] If the branch transducer does not permit inserted bifurcations then $\hat{n} = 1$ always, but this is generically not true for a more general grammar (for example, see the TKF Structure Tree model).

We require:

[2]The node $\hat{n}$ which initiates the transition to `End` is the root of the greatest active subtree of the whole guide tree (called such because $a_m = \texttt{End}\, \forall\, m \ntrianglerighteq \hat{n}$).

Nodes $m \not\trianglerighteq \hat{n}$: $a_m = \texttt{End}$.

Node $\hat{n}$: $\text{type}(a_{\hat{n}}) \in \{\texttt{Start}, \texttt{Insert}\}$

$\exists$ a transition $a_{\hat{n}} \to \texttt{End}$.

Nodes $m \triangleright \hat{n}$: $\text{type}(a_m) = \texttt{Wait}$

$\exists$ a transition $a_m \to \texttt{End}$.

In many probabilistic models, the transition from a state of type `Wait` to the `End` state has weight 1 conditional on absorbing the `End` symbol (called ϵ in formal grammar theory), but we here allow for a more general contribution to the total weight $\mathbb{F}$ of the transition.

$\hat{n}$ and Weight($\boldsymbol{a} \to \texttt{End}$) are so defined in order to ensure that there exists a direct path along which the end symbol can be passed down the tree. The grammar should be designed such that if the singlet transducer at node $\hat{n}$ can make a transition to `End`, then so can all machines at $\{m | m \trianglerighteq \hat{n}\}$. The TKF Structure Tree model satisfies this condition. A more general approach is probably possible, but it involves summing over paths $a' : a_m \to a' \to \texttt{End}$, handling possible bifurcations in these paths, etc.

References

1. Holmes I (2003) Using guide trees to construct multiple-sequence evolutionary HMMs. Bioinformatics 19 Suppl. 1: i147-157.
