# Supplementary material for: Evolutionary Triplet Models of Structured RNA
Source: PLoS Comput Biol. 2009 Aug 28;5(8):e1000483. doi: 10.1371/journal.pcbi.1000483 (PMC2725318; doi:10.1371/journal.pcbi.1000483)
Supplement: Text S2 — Algorithm for elimination of null cycles and empty bifurcations from general SCFGs (0.13 MB PDF) [file pcbi.1000483.s002.pdf]

Evolutionary triplet models of structured RNA: Text S2

Robert K. Bradley[1], Ian Holmes[1,2,*]

1 Biophysics Graduate Group, University of California, Berkeley, CA, USA

2 Department of Bioengineering, University of California, Berkeley, CA, USA

∗ E-mail: indiegram@postbox.biowiki.org

Contents

1 Exact elimination of SCFG null cycles **3**

1.1 Definitions 3

1.2 Eliminating null bifurcations 5

1.3 Eliminating null states 7

1.4 Restoring null states 8

1.5 Restoring null bifurcations 8

1 Exact elimination of SCFG null cycles

The following section describes how to transform any SCFG so as to remove null cycles while preserving access to the full posterior probability distribution over parses, including parses with null cycles.

1.1 Definitions

Let $G = (\Omega, \mathcal{N}, \mathbf{R}, P)$ be a stochastic context-free grammar (SCFG) consisting of a set of terminal symbols Ω, a set of nonterminal symbols (a.k.a. "states") $\mathcal{N}$, a set $\mathbf{R}$ of *production rules* $L \to R$ (where $L \in \mathcal{N}$ and $R \in (\mathcal{N} \cup \Omega)^*$) and a probability function on the rules, $P : (\mathcal{N} \times (\mathcal{N} \cup \Omega)^*) \to [0, \infty)$.

Let $\mathcal{L}(A)$ be the set of rules that can be applied to nonterminal $A \in \mathcal{N}$ (i.e., rules in which A appears on the left), and let $\mathcal{R}(A)$ be the set of rules that can generate nonterminal A (i.e.,rules in which A appears on the right, including bifurcations which also generate another nonterminal as well as A). Let $\mathcal{A}(A) = \mathcal{L}(A) \cup \mathcal{R}(A)$ be the set of all rules involving A. Define these also on sets of nonterminals, e.g., $\mathcal{L}(\mathbf{N}) = \bigcup_{A \in \mathbf{N}} \mathcal{L}(A)$ for $\mathbf{N} \subseteq \mathcal{N}$.

Let $\mathcal{T}$ be the set of all parse trees for G. Suppose that parse tree $T \in \mathcal{T}$ makes $n_T(\rho)$ uses of rule ρ; then define the parse tree likelihood $P(T) = \prod_{\rho \in \mathbf{R}} [P(\rho)]^{n_T(\rho)}$.

If $\sum_{T \in \mathcal{T}} P(T) = 1$, we say that G is *probabilistically normalized by parse tree.* If $\sum_{\rho \in \mathcal{L}(A)} P(\rho) = 1$ for all $A \in \mathcal{N}$, we say that G is *probabilistically normalized by production rule.* Note that normalization by production rule $\Rightarrow$ normalization by parse tree.

Let $S \in \Omega^*$ denote a terminal sequence. Let $\mathrm{seq} : \mathcal{T} \to \Omega^*$ be the function mapping a parse tree to its terminal sequence. and let $\mathrm{root} : \mathcal{T} \to \mathcal{N}$ be the function returning the root nonterminal of a parse tree. The *inside probability of S rooted at A* is $P(S|A) = \sum_{T:\mathrm{seq}(T)=S,\mathrm{root}(T)=A} P(T)$. Of particular relevance to null state elimination is the probability $P(\epsilon|A)$, where ϵ is the empty sequence. This is the probability that a nonterminal A will expire without generating any sequence.

Following earlier formalisms [1–3], we say that the grammar G has *RNA normal-form rules* if each rule in $\mathbf{R}$ takes one of the following four forms:

Termination rules with one nonterminal on the left and the empty string on the right

$$A \to \epsilon$$

Transition rules with one nonterminal on the left and the right

$$A \rightarrow B$$

Bifurcation rules with one nonterminal on the left and two on the right

$$A \rightarrow B\ C$$

Emission rules with one nonterminal on the left and right and at least one terminal on the right

$$A \rightarrow x\ B$$
$$A \rightarrow B\ y$$
$$A \rightarrow x\ B\ y$$

Here $A, B, C \in \mathcal{N}$ and $x, y \in \Omega$.

Suppose that grammar G has RNA normal-form rules. We further say that G has *RNA normal-form states* if each nonterminal (state) $A \in \mathcal{N}$ takes one of the following forms:

Null states : $\mathcal{L}(A)$ contains only transition and termination rules.

Bifurcation states : $\mathcal{L}(A)$ contains exactly one bifurcation rule, $A \rightarrow X\ Y$, where $X \neq Y$ and X, Y are both null states.

Emit states : $\mathcal{L}(A)$ contains only emission rules. Further, $\mathcal{L}(A) = \mathcal{R}(B)$ for some null state B. This null state B is called A's *post-emit* state.

Define a *null cycle* to be a nonterminal A and a sequence of rules $\rho_1, \rho_2 \ldots \rho_k$ that, when applied consecutively to A, leave A unchanged; that is, $\rho_k(\rho_{k-1} \ldots \rho_2(\rho_1(A))) = A$. Define a *null subtree* to be a null cycle using at least one bifurcation rule.

Suppose that $G = (\Omega, \mathcal{N}, \mathbf{R}, P)$ and $G' = (\Omega, \mathcal{N}', \mathbf{R}', P')$ are two grammars. We say that G and G' are *equivalent in sequence* if there is a mapping between nonterminals, $f : \mathcal{N} \to \mathcal{N}'$, such that $P(S|A) = P(S|f(A))$. We say that G and G' are *equivalent in parse* if there is a mapping between parse trees, $g : \mathcal{T} \to \mathcal{T}'$, such that $P'(T') = \sum_{T:T'=g(T)} P(T)$ and we can define

$$P(T|T') = P(T|g(T) = T') = \frac{P(T)}{P'(T')}$$

Note that equivalence in parse $\Rightarrow$ equivalence in sequence.

Suppose that a grammar G contains null cycles. We seek to transform G into a grammar G'' that is equivalent in parse and sequence, but has no null cycles. If G is normalized by parse tree, then G'' will be too; but G'' is not (necessarily) normalized by production rule.

We do the transformation in two steps, $G \to G' \to G''$. We also provide a stochastic "null cycle restoration" algorithm for sampling from $P(T|T'')$.

1.2 Eliminating null bifurcations

Note first that any SCFG can be transformed into an equivalent one with RNA normal-form states by adding null states. Without loss of generality, we therefore consider SCFGs with RNA normal-form states.

Let $G = (\Omega, \mathcal{N}, \mathbf{R}, P)$ be such an SCFG. Let $\mathbf{N} \subseteq \mathcal{N}$ be the set of null states in $\mathcal{N}$, **excluding** post-emit states. Let $\mathbf{E} \subset \mathcal{N}$ be the set of post-emit states. Let $\mathbf{B} \subseteq \mathcal{N}$ be the set of bifurcation states.

We here define the grammar G',

$$G' = (\Omega, \mathcal{N}', \mathbf{R}', P')$$

which is equivalent to G in sequence but has no null subtrees.

The new states $\mathcal{N}'$ are defined as follows. Start with $\mathcal{N}$; remove bifurcation states; then introduce a new state N' for every null $N \in \mathbf{N}$ and three new states B^0, B', B^2 for every bifurcation $B \in \mathbf{B}$.

$$\mathcal{N}' = \left(\bigcup_{N \in \mathbf{N}} \{N'\} \right) \cup \left(\bigcup_{B \in \mathbf{B}} \{B^0, B', B^2\} \right) \cup \mathcal{N} \setminus \mathbf{B}$$

The idea is that N' (in G') is a null state that is equivalent to null state N (in G) for non-empty sequence only. That is, for any terminal sequence S,

$$P'(S|N') = \begin{cases} P(S|N) & \text{if } S \neq \epsilon \\ 0 & \text{if } S = \epsilon \end{cases}$$

Similarly, B' is a null state that is equivalent to bifurcation state B for non-empty sequence, while B^2 is a null state that is is equivalent to B for parse trees where both children of B are non-empty.

In contrast, B^0 is **exactly** equivalent to B in sequence. However, null subtrees in B^0 are explicitly accounted for, their probabilities factored into the transitions $B' \to L'$ and $B' \to R'$ and the termination $B^0 \to \epsilon$, using the inside probabilities for empty sequences, $P(\epsilon|L)$ and $P(\epsilon|R)$.

The probabilities $P(\epsilon|X)$ are related by the following system of equations

$$P(\epsilon|X) = P(X \to \epsilon) + \sum_{Y} P(X \to Y) P(\epsilon|Y) + \sum_{L,R} P(X \to LR) P(\epsilon|L) P(\epsilon|R)$$

which are nonlinearly coupled (via bifurcations) but may be solved numerically, e.g., by the Newton-Raphson method, or by iterated approximation starting from a lower bound $P(\epsilon|X) \geq 0$.

The new rules and their probabilities are

$$\begin{array}{rrcl}
\forall \rho \in (\mathbf{R} \setminus \mathcal{A}(\mathbf{B})): & P'(\rho) & = & P(\rho) \\
\forall N \in \mathbf{N}: & P'(N' \to \epsilon) & = & 0 \\
\ldots \forall B \in \mathbf{B}: & P'(N' \to B') & = & P(N \to B) \\
\ldots \forall M \in \mathbf{N}: & P'(N' \to M') & = & P(N \to M) \\
\ldots \forall E \in \mathbf{E}: & P'(N' \to E) & = & P(N \to E) \\
\forall (A \to B) \in \mathcal{R}(\mathbf{B}): & P'(A \to B^0) & = & P(A \to B) \\
\forall (B \to LR) \in \mathcal{L}(\mathbf{B}): & P'(B^0 \to \epsilon) & = & P(B \to LR)\ P(\epsilon|L)\ P(\epsilon|R) \\
 & P'(B^0 \to B') & = & 1 \\
 & P'(B' \to \epsilon) & = & 0 \\
 & P'(B' \to L') & = & P(B \to LR)\ P(\epsilon|R) \\
 & P'(B' \to R') & = & P(B \to LR)\ P(\epsilon|L) \\
 & P'(B' \to B^2) & = & 1 \\
 & P'(B^2 \to L'\ R') & = & P(B \to LR)
\end{array}$$

Note that grammar G', like G, has RNA normal-form states. Note also that G' is not, in general,

normalized by production rule; however, G' is equivalent to G in parse and is therefore normalized by parse tree (if G is).

1.3 Eliminating null states

We now proceed to eliminate null cycles from G'. Since null subtrees have been eliminated, the remaining null cycles use only transition rules.

We create the grammar

$$G'' = (\Omega, \mathcal{N}', \mathbf{R}'', P'')$$

which has the same nonterminals as G', but different rules and rule probabilities.

Let $\mathbf{N}' \subseteq \mathcal{N}'$ be the set of null states in $\mathcal{N}'$, **including** post-emit states.

Define $\mathbf{t}$, the *transition matrix* of G', as $t_{XY} = P'(X \to Y)$ for all $X, Y \in \mathcal{N}'$. The effective transition probability q_{XY} between two states X, Y sums over all paths through null states (in the summand, n is the length of the null path):

$$\mathbf{q} = \sum_{n=0}^{\infty} \mathbf{t}^n = (\mathbf{1} - \mathbf{t})^{-1}$$

Here $\mathbf{1}$ is the identity matrix. The matrix inverse may be computed in the usual ways (Gauss-Jordan elimination, LU decomposition, etc.)

Since the bifurcation states of G' explicitly generate non-empty sequence, the system of equations relating the probabilities $P'(\epsilon|X)$ is completely linear and may be solved in the same way. Let $u_X = P'(\epsilon|X)$ and $v_X = P'(X \to \epsilon)$. Then

$$\mathbf{u} = \mathbf{v} + \mathbf{t}\mathbf{u}$$

whose solution is $\mathbf{u} = \mathbf{q}\mathbf{v}$. Thus $P'(\epsilon|X) = \sum_Y q_{XY} P'(Y \to \epsilon)$.

We now define P'' as follows.

- For all rules $\rho \in \mathbf{R}'$ that are **not** transitions or terminations, set $P''(\rho) = P'(\rho)$.
- For terminations from null states $X \in \mathbf{N}'$, set $P''(X \to \epsilon) = u_X$. (NB this may create some terminations $X \to \epsilon$ that were not present in G'.)
- For transitions from null states $X \in \mathbf{N}'$ to non-null states $Y \notin \mathbf{N}'$, set $P''(X \to Y) = q_{XY}$. (NB

this may create some transitions $X \to Y$ that were not present in G'.)

- For transitions **to** null states $X \in \mathbf{N}'$, set $P''(A \to X) = 0$.

Although we have left null states in G'', they now have no incoming transitions and are inaccessible unless they are post-bifurcation states (i.e.,states which appear on the right-hand side of bifurcation rules), post-emit states, or the start (root) state. All other null states can therefore be dropped from $\mathcal{N}''$.

1.4 Restoring null states

Suppose that ρ'' is a rule in G''. Algorithm 1 samples from the distribution of equivalent parse subtrees in G' (possibly containing null cycles). In order to sample from $P(T'|T'')$ we need simply map Algorithm 1 to each rule in T''.

For parameter estimation by Expectation Maximization and some other applications, it is useful to know the expected number of times that a transition was used, summed over the posterior distribution of parse trees (including those with null cycles). If $n''(\rho'')$ is the expected number of times that transition ρ'' was used according to an Inside-Outside computation on G'', then the corresponding expectations $n'(\rho')$ are given by

$$\begin{aligned} n'(X \to Y) &= n''(X \to Y)\frac{P''(X \to Y)}{q_{XY}} + \sum_Z n''(X \to Z)\frac{P''(X \to Y) q_{YZ}}{q_{XZ}} \\ n'(X \to \epsilon) &= n''(X \to \epsilon)\frac{P''(X \to \epsilon)}{P''(\epsilon|X)} + \sum_W n''(W \to \epsilon)\frac{q_{WX} P''(X \to \epsilon)}{P''(\epsilon|W)} \end{aligned}$$

Expectations for other rules (bifurcations and emissions) are the same for G' as G''.

1.5 Restoring null bifurcations

Suppose that ρ' is a rule in G'. Algorithm 2 samples from the distribution of equivalent parse subtrees in G (possibly containing null subtrees). This algorithm also calls Algorithm 3, which samples from the distribution of empty subtrees rooted at a particular nonterminal. In order to sample from $P(T|T')$ we need simply map Algorithm 2 to each rule in T'.

If $n'(\rho')$ is the expected number of times that rule ρ' was used by G', then the corresponding expectations $n(\rho)$ are given by

$$\begin{aligned} n(B \to L\ R) &= n'(B' \to L') + n'(B' \to R') + n'(B^0 \to L'\ R') + d(B \to L\ R) \\ n(X \to Y) &= n'(X \to Y) + d(X \to Y) \\ n(X \to \epsilon) &= n'(X \to \epsilon) + d(X \to \epsilon) \end{aligned}$$

Expectations for emissions are the same for G as G'. In the above expressions $d(\rho)$ is the expected usage of rule ρ by null subtrees:

$$d(\rho) = \sum_{(B \to LR) \in \mathbf{R}} \left[n'(B' \to L') c_R(\rho) + n'(B' \to R') c_L(\rho) + n'(B^0 \to \epsilon)(c_L(\rho) + c_R(\rho)) \right]$$

where $c_W(\rho)$ is the expected usage of rule ρ by an empty parse tree rooted at W, given by

$$\begin{aligned} c_X(\rho) P(\epsilon|X) &= P(X \to \epsilon)\delta_{\rho=(X \to \epsilon)} + \sum_Y P(X \to Y) P(\epsilon|Y) \left(c_Y(\rho) + \delta_{\rho=(X \to Y)} \right) \\ &\quad + \sum_{L,R} P(X \to LR) P(\epsilon|L) P(\epsilon|R) \left(c_L(\rho) + c_R(\rho) + \delta_{\rho=(X \to LR)} \right) \end{aligned}$$

where δ_U is the Kronecker delta (1 if condition U is true, 0 if it is false). Note that in contrast to the system of equations for $P(\epsilon|X)$, this is a linear system of equations of the form $\mathbf{c} = \mathbf{M}\mathbf{c} + \mathbf{k}$, which may be solved by matrix inversion: $\mathbf{c} = (\mathbf{1} - \mathbf{M})^{-1}\mathbf{k}$.

References

1. Durbin R, Eddy S, Krogh A, Mitchison G (1998) Biological Sequence Analysis: Probabilistic Models of Proteins and Nucleic Acids. Cambridge, UK: Cambridge University Press.

2. Holmes I, Rubin GM (2002) Pairwise RNA structure comparison using stochastic context-free grammars. Pacific Symposium on Biocomputing, 2002.

3. Holmes I (2005) Accelerated probabilistic inference of RNA structure evolution. BMC Bioinformatics 6.

Algorithms

Input: Rule $\rho'' \in \mathbf{R}''$
Output: Parse (sub)tree $T' \in \mathcal{T}'$
switch ρ'' **do**
  case $X \to Y$
    if `Random[0,1]` $< P(X \to Y)/q_{XY}$ **then**
      return $(X \to Y)$;
    else
      Let $z = \sum_W q_{XW} P'(W \to Y)$;
      Sample state V from probability distribution $P(V) = q_{XV} P'(V \to Y)/z$;
      return (restoreTransitions($X \to V$) $\to Y$) ;
    end
  end
  case $X \to \epsilon$
    if `Random[0,1]` $< P'(X \to \epsilon)/P'(\epsilon|X)$ **then**
      return $(X \to \epsilon)$;
    else
      Let $z = \sum_W q_{XW} P'(W \to \epsilon)$;
      Sample state V from probability distribution $P(V) = q_{XV} P'(V \to \epsilon)/z$;
      return (restoreTransitions($X \to V$) $\to \epsilon$) ;
    end
  end
  otherwise
    return (ρ'') ;
  end
end

Algorithm 1: Subroutine restoreTransitions(ρ'') for the null cycle elimination procedure.

```
Input: Rule ρ′ ∈ R′
Output: Parse (sub)tree T ∈ 𝒯
switch ρ′ do
    case B⁰ → ε
        Let B → L R be the original bifurcation in R ;
        T_L ← sampleNullSubtree(L) ;
        T_R ← sampleNullSubtree(R) ;
        return (B → T_L T_R) ;
    end
    case B′ → L′
        Let B → L R be the original bifurcation in R ;
        T_R ← sampleNullSubtree(R) ;
        return (B → L T_R) ;
    end
    case B′ → R′
        Let B → L R be the original bifurcation in R ;
        T_L ← sampleNullSubtree(L) ;
        return (B → T_L R) ;
    end
    case B² → L′ R′
        Let B → L R be the original bifurcation in R ;
        return (B → L R) ;
    end
    case B⁰ → B′  return () ;
    case B′ → B²  return () ;
    otherwise
        Let ρ be the original rule in R ;
        return (ρ) ;
    end
end
```

Algorithm 2: Subroutine restoreBifurcations(ρ') for the null cycle elimination procedure.

```
Input: Nonterminal A ∈ 𝒩
Output: Parse tree T ∈ 𝒯 : seq(T) = ε, root(T) = A
if A is a bifurcation state, A → X Y, then
    T_X ← sampleNullSubtree(X) ;
    T_Y ← sampleNullSubtree(Y) ;
    return (A → T_X T_Y) ;
else
    if Random[0,1] < P(A → ε)/P(ε|A) then
        return (A → ε) ;
    else
        Let z = Σ_Y P(A → Y)P(ε|Y) ;
        Sample state X from probability distribution P(X) = P(A → X)P(ε|X)/z ;
        T_X ← sampleNullSubtree(X) ;
        return (A → T_X) ;
    end
end
```

Algorithm 3: Subroutine sampleNullSubtree(A) for the null cycle elimination procedure.
