# Supplementary material for: Evolutionary Triplet Models of Structured RNA
Source: PLoS Comput Biol. 2009 Aug 28;5(8):e1000483. doi: 10.1371/journal.pcbi.1000483 (PMC2725318; doi:10.1371/journal.pcbi.1000483)
Supplement: Text S3 — Description of software tools (0.09 MB PDF) [file pcbi.1000483.s003.pdf]

Evolutionary triplet models of structured RNA: Text S3

Robert K. Bradley[1], Ian Holmes[1,2,*]

1 Biophysics Graduate Group, University of California, Berkeley, CA, USA

2 Department of Bioengineering, University of California, Berkeley, CA, USA

* E-mail: indiegram@postbox.biowiki.org

1 Software

All tools are available from `http://biowiki.org/dart` as part of the DART software package for sequence analysis.

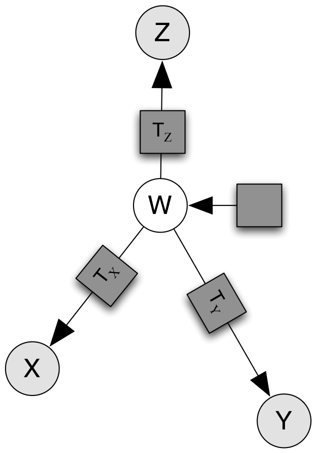

Figure 1. Transducer composition on a star phylogeny with three (extant) leaf sequences. An ancestral sequence W evolves into three descendant sequences X, Y and Z. A singlet transducer (the horizontal gray box) emits an ancestral sequence and structure and three branch transducers (the gray boxes labeled T_X, T_Y and T_Z) mutate it according to the specified evolutionary model. Gray nodes correspond to observed data and white nodes unobserved data. If the branch transducers are time-reversible, then this star phylogeny with three leaves is the neighborhood of any interior node in a (binary) phylogenetic tree, from which it follows that evaluating the likelihood function on this star phylogeny is sufficient, in principle, for a sampling algorithm on an arbitrary phylogeny.

1.1 Automated grammar construction

We implemented our model construction algorithm on the three-taxon star phylogeny (Figure 1) in a set of Perl scripts. Given a singlet transducer modeling ancestral sequences and structures and a branch transducer modeling structural evolution, the scripts generate C++ code to create the corresponding jointly-normalized SCFG. All possible models of structural evolution which can be represented by a Pair SCFG are permitted as input to the scripts, allowing for flexible and automated model design.

Given files describing the singlet and branch transducers, including weights of all transitions which may be functions of evolutionary distance, the package ComposedTreeTransducer::FourWayComposedTT can automatically generate the state graph and transition matrix of a multi-sequence model of three extant sequences. It removes the useless windback `Null` states described in the paper and introduces

effective direct transitions caused by bifurcations with possibly-empty children. The package ComposedTreeTransducer::TripletSCFG transforms a multi-sequence model created by ComposedTreeTransducer::FourWayComposedTT into the corresponding jointly-normalized three-sequence SCFG and generates C++ code to build the model.

Example singlet and branch transducers files are provided for a simple Pair HMM model, a simple Pair SCFG model and the full TKF Structure Tree model.

1.2 Reconstruction of ancestral structures

The program Indiegram can perform maximum-likelihood inference on the three-sequence SCFGs automatically generated by the ComposedTreeTransducer::TripletSCFG package. Complete or no structural information for the three extant sequences can be supplied as input.

1.3 Simulation of RNA family evolution

The program that we wrote to simulate RNA structural alignments from the TKFST model, `evolsayer.pl`, is available as part of the same software distribution as Indiegram (the DART package). Another script (`animate-evolsayer.pl`) can be used to make animations of the evolving RNA structures, in combination with the RNAplot program in the ViennaRNA package [1].

References

1. Hofacker IL, Fontana W, Stadler PF, Bonhoeffer S, Tacker M, et al. (1994) Fast folding and comparison of RNA secondary structures. Monatshefte für Chemie 125: 167-188.
