# Supplementary material for: Evolutionary Triplet Models of Structured RNA
Source: PLoS Comput Biol. 2009 Aug 28;5(8):e1000483. doi: 10.1371/journal.pcbi.1000483 (PMC2725318; doi:10.1371/journal.pcbi.1000483)
Supplement: Text S4 — Example presentation of TKFST inference grammars in Indiegram format (0.14 MB PDF) [file pcbi.1000483.s004.pdf]

Evolutionary triplet models of structured RNA: Text S4

Robert K. Bradley[1], Ian Holmes[1,2,*]

1 Biophysics Graduate Group, University of California, Berkeley, CA, USA

2 Department of Bioengineering, University of California, Berkeley, CA, USA

∗ E-mail: indiegram@postbox.biowiki.org

1 Inference grammars for the TKFST model

This section describes multi-sequence grammars that can be used to infer alignments, structures and ancestors under the TKF Structure Tree (TKFST) model.

The TKFST model on a two-branch phylogeny

While the idea of composing conditionally-normalized models on a tree is intuitive, the resulting models can quickly become too complex to deal with by hand, necessitating an algorithm for automated model construction. We constructed the model corresponding to TKFST on the simple the two-branch phylogeny $a \xrightarrow{t} b \xrightarrow{t'} c$. The results, shown below, make clear that by-hand model construction quickly becomes impractical as model complexity or tree size increases.

We explicitly note cases where certain sets of grammar rules can be derived "automatically" from simpler sets, motivating the development of our generic tree transducer composition algorithm.

Time-dependent probabilities

Here $n \in \{l, s\}$ denotes the type of structural element (loop or stem). $\mathbf{R}^{(n)}$ is the rate matrix and π_n the equilibrium distribution. λ_n is the insertion rate and μ_n is the deletion rate.

$$
\begin{aligned}
\alpha_n(t) &= \exp(-\mu_n t) \\
\beta_n(t) &= \frac{\lambda_n(1 - \exp((\lambda_n - \mu_n)t))}{\mu_n - \lambda_n \exp((\lambda_n - \mu_n)t)} \\
\gamma_n(t) &= 1 - \frac{\mu_n(1 - \exp((\lambda_n - \mu_n)t))}{(1 - \exp(-\mu_n t))(\mu_n - \lambda_n \exp((\lambda_n - \mu_n)t))} \\
\kappa_n(t) &= \lambda_n / \mu_n \\
M_n(i, j; t) &= \exp(\mathbf{R}^{(n)} t)_{ij}
\end{aligned}
$$

Let $\alpha_n \equiv \alpha_n(t)$ and $\alpha'_n = \alpha_n(t')$; similarly $\beta'_n, \gamma'_n, M'_n$.

Grammars

Note that any of the grammars can be "downsized" to a smaller number of sequences, simply by dropping terminals, yielding alternative applications for each grammar.

For example, the Pair SCFG for $a \xrightarrow{t} b$ becomes a Single SCFG for a if sequence b is dropped by removing terminals w, x. Stochastic traceback then can be used to sample the descendant b. This corresponds to a forward-time simulator when we apply the Inside & stochastic traceback algorithms.

Similarly, $a \xrightarrow{t} b \xrightarrow{t'} c$ can be viewed as a pair grammar $a \xrightarrow{t+t'} c$ that can be used to sample an unknown evolutionary intermediate b. Again, this requires only the Inside & stochastic traceback algorithms.

Singlet rules. This section contains three singlet grammars, one for each of the sequences a, b and c.

Note that Tables 2 and 3 may be deduced automatically from Table 1 if it is known that the underlying process is initially at equilibrium.

Pair rules. This section contains two pairwise rule-sets.

The pair grammar for $a \xrightarrow{t} b$ is the union of Tables 1, 2 and 4.

The pair grammar for $b \xrightarrow{t'} c$ is the union of Tables 2, 3 and 5.

Note that Table 5 may be deduced automatically from Table 4 if it is known that the underlying process is stationary.

Triplet rules. Because of its length, we have split the triplet rule-set over three tables:

Table 6: Rules for transforming $\{L_{abc}, S_{abc}\}$, after emissions to c;

Table 7: Rules for transforming $\{L_{ab*c}, S_{ab*c}\}$, after deletions on $b \xrightarrow{t'} c$;

Table 8: Rules for transforming $\{L_{a*bc}, S_{a*bc}\}$, after deletions on $a \xrightarrow{t} b$.

The triplet grammar for $a \xrightarrow{t} b \xrightarrow{t'} c$ is the union of Tables 1 through 8.

Note that Tables 6 through 8 may be deduced automatically from Tables 1 through 5, **regardless of the properties of the underlying process,** using the tree transducer composition algorithm.

TKF Structure Tree singlet rules (a)
Sequence a terminals: $\{u, v\}$.

lhs	$\to$	rhs	$P(a)$
L_a	$\to$	$u\ L_a$	$\kappa_l \pi_l(u)$
	$\mid$	$S_a\ L_a$	$\kappa_l \pi_l(S)$
	$\mid$	ϵ	$1 - \kappa_l$
S_a	$\to$	$u\ S_a\ v$	$\kappa_s \pi_s(uv)$
	$\mid$	L_a	$1 - \kappa_s$

Table 1. Singlet rule-set for a.

TKF Structure Tree singlet rules (b)
Sequence b terminals: $\{w, x\}$.

lhs	$\to$	rhs	$P(b)$
L_b	$\to$	$w\ L_b$	$\kappa_l \pi_l(w)$
	$\mid$	$S_b\ L_b$	$\kappa_l \pi_l(S)$
	$\mid$	ϵ	$1 - \kappa_l$
S_b	$\to$	$w\ S_b\ x$	$\kappa_s \pi_s(wx)$
	$\mid$	L_b	$1 - \kappa_s$

Table 2. Singlet rule-set for b.

TKF Structure Tree singlet rules (c)
Sequence c terminals: $\{y, z\}$.

lhs	$\to$	rhs	$P(c)$
L_c	$\to$	$y\ L_c$	$\kappa_l \pi_l(y)$
	$\mid$	$S_c\ L_c$	$\kappa_l \pi_l(S)$
	$\mid$	ϵ	$1 - \kappa_l$
S_c	$\to$	$y\ S_c\ z$	$\kappa_s \pi_s(yz)$
	$\mid$	L_c	$1 - \kappa_s$

Table 3. Singlet rule-set for c.

TKF Structure Tree pair rules ($a \xrightarrow{t} b$)
Sequence a terminals: $\{u, v\}$. *Sequence b terminals:* $\{w, x\}$.

lhs	→	rhs	$P(a)$	$P(b\|a)$
L_{ab}	$\rightarrow$	$u\ w\ L_{ab}$	$\kappa_l \pi_l(u)$	$(1-\beta_l)\alpha_l M_l(u,w)$
	$\|$	$w\ L_{ab}$	1	$\beta_l \pi_l(w)$
	$\|$	$u\ L_{a*b}$	$\kappa_l \pi_l(u)$	$(1-\beta_l)(1-\alpha_l)$
	$\|$	$S_{ab}\ L_{ab}$	$\kappa_l \pi_l(S)$	$(1-\beta_l)\alpha_l$
	$\|$	$S_b\ L_{ab}$	1	$\beta_l \pi_l(S)$
	$\|$	$S_a\ L_{a*b}$	$\kappa_l \pi_l(S)$	$(1-\beta_l)(1-\alpha_l)$
	$\|$	ϵ	$1-\kappa_l$	$1-\beta_l$
S_{ab}	$\rightarrow$	$u\ w\ S_{ab}\ x\ v$	$\kappa_s \pi_s(uv)$	$(1-\beta_s)\alpha_s M_s(uv,wx)$
	$\|$	$w\ S_{ab}\ x$	1	$\beta_s \pi_s(wx)$
	$\|$	$u\ S_{a*b}\ v$	$\kappa_s \pi_s(uv)$	$(1-\beta_s)(1-\alpha_s)$
	$\|$	L_{ab}	$1-\kappa_s$	$1-\beta_s$
L_{a*b}	$\rightarrow$	$u\ w\ L_{ab}$	$\kappa_l \pi_l(u)$	$(1-\gamma_l)\alpha_l M_l(u,w)$
	$\|$	$w\ L_{ab}$	1	$\gamma_l \pi_l(w)$
	$\|$	$u\ L_{a*b}$	$\kappa_l \pi_l(u)$	$(1-\gamma_l)(1-\alpha_l)$
	$\|$	$S_{ab}\ L_{ab}$	$\kappa_l \pi_l(S)$	$(1-\gamma_l)\alpha_l$
	$\|$	$S_b\ L_{ab}$	1	$\gamma_l \pi_l(S)$
	$\|$	$S_a\ L_{a*b}$	$\kappa_l \pi_l(S)$	$(1-\gamma_l)(1-\alpha_l)$
	$\|$	ϵ	$1-\kappa_l$	$1-\gamma_l$
S_{a*b}	$\rightarrow$	$u\ w\ S_{ab}\ x\ v$	$\kappa_s \pi_s(uv)$	$(1-\gamma_s)\alpha_s M_s(uv,wx)$
	$\|$	$w\ S_{ab}\ x$	1	$\gamma_s \pi_s(wx)$
	$\|$	$u\ S_{a*b}\ v$	$\kappa_s \pi_s(uv)$	$(1-\gamma_s)(1-\alpha_s)$
	$\|$	L_{ab}	$1-\kappa_s$	$1-\gamma_s$

Table 4. Pair rule-set for $a \xrightarrow{t} b$ branch. Requires Table 1 and Table 2.

TKF Structure Tree pair rules ($b \xrightarrow{t'} c$)
Sequence b *terminals:* $\{w, x\}$. *Sequence* c *terminals:* $\{y, z\}$.

lhs	→	rhs	$P(b)$	$P(c\|b)$
L_{bc}	$\rightarrow$	$w\ y\ L_{bc}$	$\kappa_l \pi_l(w)$	$(1-\beta'_l)\alpha'_l M'_l(w,y)$
	$\|$	$y\ L_{bc}$	1	$\beta'_l \pi_l(y)$
	$\|$	$w\ L_{b*c}$	$\kappa_l \pi_l(w)$	$(1-\beta'_l)(1-\alpha'_l)$
	$\|$	$S_{bc}\ L_{bc}$	$\kappa_l \pi_l(S)$	$(1-\beta'_l)\alpha'_l$
	$\|$	$S_c\ L_{bc}$	1	$\beta'_l \pi_l(S)$
	$\|$	$S_b\ L_{b*c}$	$\kappa_l \pi_l(S)$	$(1-\beta'_l)(1-\alpha'_l)$
	$\|$	ϵ	$1-\kappa_l$	$1-\beta'_l$
S_{bc}	$\rightarrow$	$w\ y\ S_{bc}\ z\ x$	$\kappa_s \pi_s(wx)$	$(1-\beta'_s)\alpha'_s M'_s(wx,yz)$
	$\|$	$y\ S_{bc}\ z$	1	$\beta'_s \pi_s(yz)$
	$\|$	$w\ S_{b*c}\ x$	$\kappa_s \pi_s(wx)$	$(1-\beta'_s)(1-\alpha'_s)$
	$\|$	L_{bc}	$1-\kappa_s$	$1-\beta'_s$
L_{b*c}	$\rightarrow$	$w\ y\ L_{bc}$	$\kappa_l \pi_l(w)$	$(1-\gamma'_l)\alpha'_l M'_l(w,y)$
	$\|$	$y\ L_{bc}$	1	$\gamma'_l \pi_l(y)$
	$\|$	$w\ L_{b*c}$	$\kappa_l \pi_l(w)$	$(1-\gamma'_l)(1-\alpha'_l)$
	$\|$	$S_{bc}\ L_{bc}$	$\kappa_l \pi_l(S)$	$(1-\gamma'_l)\alpha'_l$
	$\|$	$S_c\ L_{bc}$	1	$\gamma'_l \pi_l(S)$
	$\|$	$S_b\ L_{b*c}$	$\kappa_l \pi_l(S)$	$(1-\gamma'_l)(1-\alpha'_l)$
	$\|$	ϵ	$1-\kappa_l$	$1-\gamma'_l$
S_{b*c}	$\rightarrow$	$w\ y\ S_{bc}\ z\ x$	$\kappa_s \pi_s(wx)$	$(1-\gamma'_s)\alpha'_s M'_s(wx,yz)$
	$\|$	$y\ S_{bc}\ z$	1	$\gamma'_s \pi_s(yz)$
	$\|$	$w\ S_{b*c}\ x$	$\kappa_s \pi_s(wx)$	$(1-\gamma'_s)(1-\alpha'_s)$
	$\|$	L_{bc}	$1-\kappa_s$	$1-\gamma'_s$

Table 5. Pair rule-set for $b \xrightarrow{t'} c$ branch. Requires Table 2 and Table 3.

TKF Structure Tree triplet rules ($a \xrightarrow{t} b \xrightarrow{t'} c$): "abc" states (post emission at c)
Sequence a terminals: $\{u, v\}$. Sequence b terminals: $\{w, x\}$. Sequence c terminals: $\{y, z\}$.

lhs	→	rhs	$P(a)$	$P(b\|a)$	$P(c\|b)$
L_{abc}	$\rightarrow$	$u\ w\ y\ L_{abc}$	$\kappa_l \pi_l(u)$	$(1-\beta_l)\alpha_l M_l(u,w)$	$(1-\beta'_l)\alpha'_l M'_l(w,y)$
	|	$y\ L_{abc}$	1	1	$\beta'_l \pi_l(y)$
	|	$w\ y\ L_{abc}$	1	$\beta_l \pi_l(w)$	$(1-\beta'_l)\alpha'_l M'_l(w,y)$
	|	$w\ L_{ab*c}$	1	$\beta_l \pi_l(w)$	$(1-\beta'_l)(1-\alpha'_l)$
	|	$u\ w\ L_{ab*c}$	$\kappa_l \pi_l(u)$	$(1-\beta_l)\alpha_l M_l(u,w)$	$(1-\beta'_l)(1-\alpha'_l)$
	|	$u\ L_{a*bc}$	$\kappa_l \pi_l(u)$	$(1-\beta_l)(1-\alpha_l)$	$1-\beta'_l$
	|	$S_{abc}\ L_{abc}$	$\kappa_l \pi_l(S)$	$(1-\beta_l)\alpha_l$	$(1-\beta'_l)\alpha'_l$
	|	$S_c\ L_{abc}$	1	1	$\beta'_l \pi_l(S)$
	|	$S_{bc}\ L_{abc}$	1	$\beta_l \pi_l(S)$	$(1-\beta'_l)\alpha'_l$
	|	$S_b\ L_{ab*c}$	1	$\beta_l \pi_l(S)$	$(1-\beta'_l)(1-\alpha'_l)$
	|	$S_{ab}\ L_{ab*c}$	$\kappa_l \pi_l(S)$	$(1-\beta_l)\alpha_l$	$(1-\beta'_l)(1-\alpha'_l)$
	|	$S_a\ L_{a*bc}$	$\kappa_l \pi_l(S)$	$(1-\beta_l)(1-\alpha_l)$	$1-\beta'_l$
	|	ϵ	$1-\kappa_l$	$1-\beta_l$	$1-\beta'_l$
S_{abc}	$\rightarrow$	$u\ w\ y\ S_{abc}\ z\ x\ v$	$\kappa_s \pi_s(uv)$	$(1-\beta_s)\alpha_s M_s(uv,wx)$	$(1-\beta'_s)\alpha'_s M'_s(wx,yz)$
	|	$y\ S_{abc}\ z$	1	1	$\beta'_s \pi_s(yz)$
	|	$w\ y\ S_{abc}\ z\ x$	1	$\beta_s \pi_s(wx)$	$(1-\beta'_s)\alpha'_s M'_s(wx,yz)$
	|	$w\ S_{ab*c}\ x$	1	$\beta_s \pi_s(wx)$	$(1-\beta'_s)(1-\alpha'_s)$
	|	$u\ w\ S_{ab*c}\ x\ v$	$\kappa_s \pi_s(uv)$	$(1-\beta_s)\alpha_s M_s(uv,wx)$	$(1-\beta'_s)(1-\alpha'_s)$
	|	$u\ S_{a*bc}\ v$	$\kappa_s \pi_s(uv)$	$(1-\beta_s)(1-\alpha_s)$	$1-\beta'_s$
	|	L_{abc}	$1-\kappa_s$	$1-\beta_s$	$1-\beta'_s$

Table 6. Triplet rule-set for "*abc*" states (post emission at c) on $a \xrightarrow{t} b \xrightarrow{t'} c$ tree. Requires Tables 1 through 8.

TKF Structure Tree triplet rules ($a \xrightarrow{t} b \xrightarrow{t'} c$): "$ab*c$" states (post deletion on $b \xrightarrow{t'} c$ branch)
Sequence a terminals: $\{u, v\}$. Sequence b terminals: $\{w, x\}$. Sequence c terminals: $\{y, z\}$.

lhs	→	rhs	$P(a)$	$P(b\|a)$	$P(c\|b)$
L_{ab*c}	→	$u\ w\ y\ L_{abc}$	$\kappa_l \pi_l(u)$	$(1-\beta_l)\alpha_l M_l(u,w)$	$(1-\gamma'_l)\alpha'_l M'_l(w,y)$
	|	$y\ L_{abc}$	1	1	$\gamma'_l \pi_l(y)$
	|	$w\ y\ L_{abc}$	1	$\beta_l \pi_l(w)$	$(1-\gamma'_l)\alpha'_l M'_l(w,y)$
	|	$w\ L_{ab*c}$	1	$\beta_l \pi_l(w)$	$(1-\gamma'_l)(1-\alpha'_l)$
	|	$u\ w\ L_{ab*c}$	$\kappa_l \pi_l(u)$	$(1-\beta_l)\alpha_l M_l(u,w)$	$(1-\gamma'_l)(1-\alpha'_l)$
	|	$u\ L_{a*bc}$	$\kappa_l \pi_l(u)$	$(1-\beta_l)(1-\alpha_l)$	$1-\gamma'_l$
	|	$S_{abc}\ L_{abc}$	$\kappa_l \pi_l(S)$	$(1-\beta_l)\alpha_l$	$(1-\gamma'_l)\alpha'_l$
	|	$S_c\ L_{abc}$	1	1	$\gamma'_l \pi_l(S)$
	|	$S_{bc}\ L_{abc}$	1	$\beta_l \pi_l(S)$	$(1-\gamma'_l)\alpha'_l$
	|	$S_b\ L_{ab*c}$	1	$\beta_l \pi_l(S)$	$(1-\gamma'_l)(1-\alpha'_l)$
	|	$S_{ab}\ L_{ab*c}$	$\kappa_l \pi_l(S)$	$(1-\beta_l)\alpha_l$	$(1-\gamma'_l)(1-\alpha'_l)$
	|	$S_a\ L_{a*bc}$	$\kappa_l \pi_l(S)$	$(1-\beta_l)(1-\alpha_l)$	$1-\gamma'_l$
	|	ϵ	$1-\kappa_l$	$1-\beta_l$	$1-\gamma'_l$
S_{ab*c}	→	$u\ w\ y\ S_{abc}\ z\ x\ v$	$\kappa_s \pi_s(uv)$	$(1-\beta_s)\alpha_s M_s(uv,wx)$	$(1-\gamma'_s)\alpha'_s M'_s(wx,yz)$
	|	$y\ S_{abc}\ z$	1	1	$\gamma'_s \pi_s(yz)$
	|	$w\ y\ S_{abc}\ z\ x$	1	$\beta_s \pi_s(wx)$	$(1-\gamma'_s)\alpha'_s M'_s(wx,yz)$
	|	$w\ S_{ab*c}\ x$	1	$\beta_s \pi_s(wx)$	$(1-\gamma'_s)(1-\alpha'_s)$
	|	$u\ w\ S_{ab*c}\ x\ v$	$\kappa_s \pi_s(uv)$	$(1-\beta_s)\alpha_s M_s(uv,wx)$	$(1-\gamma'_s)(1-\alpha'_s)$
	|	$u\ S_{a*bc}\ v$	$\kappa_s \pi_s(uv)$	$(1-\beta_s)(1-\alpha_s)$	$1-\gamma'_s$
	|	L_{abc}	$1-\kappa_s$	$1-\beta_s$	$1-\gamma'_s$

Table 7. Triplet rule-set for "$ab*c$" states (post deletion on $b \xrightarrow{t'} c$ branch) on $a \xrightarrow{t} b \xrightarrow{t'} c$ tree. Requires Tables 1 through 8.

TKF Structure Tree triplet rules ($a \xrightarrow{t} b \xrightarrow{t'} c$)*: "*$a * bc$*" states (post deletion on* $a \xrightarrow{t} b$ *branch)*
Sequence a terminals: $\{u, v\}$. *Sequence b terminals:* $\{w, x\}$. *Sequence c terminals:* $\{y, z\}$.

lhs	$\rightarrow$	rhs	$P(a)$	$P(b\|a)$	$P(c\|b)$
L_{a*bc}	$\rightarrow$	$u\ w\ y\ L_{abc}$	$\kappa_l \pi_l(u)$	$(1-\gamma_l)\alpha_l M_l(u,w)$	$\alpha'_l M'_l(w,y)$
	$\|$	$w\ y\ L_{abc}$	1	$\gamma_l \pi_l(w)$	$\alpha'_l M'_l(w,y)$
	$\|$	$w\ L_{ab*c}$	1	$\gamma_l \pi_l(w)$	$1-\alpha'_l$
	$\|$	$u\ w\ L_{ab*c}$	$\kappa_l \pi_l(u)$	$(1-\gamma_l)\alpha_l M_l(u,w)$	$1-\alpha'_l$
	$\|$	$u\ L_{a*bc}$	$\kappa_l \pi_l(u)$	$(1-\gamma_l)(1-\alpha_l)$	1
	$\|$	$S_{abc}\ L_{abc}$	$\kappa_l \pi_l(S)$	$(1-\gamma_l)\alpha_l$	α'_l
	$\|$	$S_{bc}\ L_{abc}$	1	$\gamma_l \pi_l(S)$	α'_l
	$\|$	$S_b\ L_{ab*c}$	1	$\gamma_l \pi_l(S)$	$1-\alpha'_l$
	$\|$	$S_{ab}\ L_{ab*c}$	$\kappa_l \pi_l(S)$	$(1-\gamma_l)\alpha_l$	$1-\alpha'_l$
	$\|$	$S_a\ L_{a*bc}$	$\kappa_l \pi_l(S)$	$(1-\gamma_l)(1-\alpha_l)$	1
	$\|$	ϵ	$1-\kappa_l$	$1-\gamma_l$	1
S_{a*bc}	$\rightarrow$	$u\ w\ y\ S_{abc}\ z\ x\ v$	$\kappa_s \pi_s(uv)$	$(1-\gamma_s)\alpha_s M_s(uv,wx)$	$\alpha'_s M'_s(wx,yz)$
	$\|$	$w\ y\ S_{abc}\ z\ x$	1	$\gamma_s \pi_s(wx)$	$\alpha'_s M'_s(wx,yz)$
	$\|$	$w\ S_{ab*c}\ x$	1	$\gamma_s \pi_s(wx)$	$1-\alpha'_s$
	$\|$	$u\ w\ S_{ab*c}\ x\ v$	$\kappa_s \pi_s(uv)$	$(1-\gamma_s)\alpha_s M_s(uv,wx)$	$1-\alpha'_s$
	$\|$	$u\ S_{a*bc}\ v$	$\kappa_s \pi_s(uv)$	$(1-\gamma_s)(1-\alpha_s)$	1
	$\|$	L_{abc}	$1-\kappa_s$	$1-\gamma_s$	1

Table 8. Triplet rule-set for "$a * bc$" states (post deletion on $a \xrightarrow{t} b$ branch) on $a \xrightarrow{t} b \xrightarrow{t'} c$ tree. Requires Tables 1 through 8.

2 The TKF Structure Tree model as a transducer

We can cast the TKF Structure Tree (TKFST) model as a transducer. This means we can automatically deduce rules like those shown for the TKFST model in Section 1.

2.1 The single-sequence TKFST model as a singlet transducer

The states of the singlet transducer are shown in Table 9. Allowed transitions between these states are shown in Section 1.

state	type	absorb	emit	$e(\bullet\|\text{TKFST})$
L	`Start`			
I_L	`Insert`		(x, null)	$p(x)$
S	`Start`			
I_S	`Insert`		(x, y)	$p(x, y)$
B	`Insert`		$(L\,S)$	1

Table 9. States of the singlet transducer of the TKF Structure Tree model [1]. Singlet transducers can only have states of type `Start` or `Insert`. This is the indiegram-style transducer equivalent of the singlet TKFST SCFG in Section 1.

2.2 The two-sequence TKFST model as a branch transducer

The states of the branch transducer are shown in Table 10. Allowed transitions between these states are shown in Section 1.

2.3 The multi-sequence TKFST model as a composite transducer

We can use the state graph construction algorithm described in the paper and detailed in Text S1 to create a model of the simultaneous evolution of several sequences.

Consider the simple model shown in Figure 1. The state of the multi-sequence model describing this model is a 3-vector $\boldsymbol{a} = (a_1, a_2, a_3)$, where a_1 is the state of the singlet transducer generating the ancestral sequence W and a_2 and a_3 are the states of the branch transducers evolving W into extant sequences X and Y.

We can show some of the allowed transitions of this multi-sequence model. The state of the branch transducer associated with the active node n is shown in bold.

state	type	absorb	emit	$e(\bullet \mid \mathrm{TKFST})$
L	`Start`			
I_L	`Insert`		(u, null)	$p(u)$
M_L	`Match`	(x, null)	(u, null)	$p(u \mid x)$
D_L	`Match`	(x, null)	$(\mathrm{GAP}, \mathrm{null})$	1
W_L	`Wait`			
S	`Start`			
I_S	`Insert`		(u, v)	$p(u, v)$
M_S	`Match`	(x, y)	(u, v)	$p(u, v \mid x, y)$
D_S	`Match`	(x, y)	$(\mathrm{GAP}, \mathrm{GAP})$	1
W_S	`Wait`			
B_i	`Insert`		$(L_i\, S_i)$	1
B	`Match`	$(L\, S)$	$(L\, S)$	1
B_p	`Match`	$(L\, S)$	$(L$ `End`$)$	1
B_e	`Match`	$(L$ `End`$)$	$(L$ `End`$)$	1

Table 10. States of the branch transducer of the TKF Structure Tree model [1]. States which have the same names as states of the singlet transducer in Table 9 are the branch equivalent of the corresponding singlet states (e.g. a `Match` state might be the branch equivalent of an `Insert` state). States L_i and S_i are the `Start` states of a sub-model (not shown) identical in structure to the singlet transducer. They are used to insert a new stem-loop structure. This is the indiegram-style transducer equivalent of the conditional pair TKFST SCFG in Section 1.

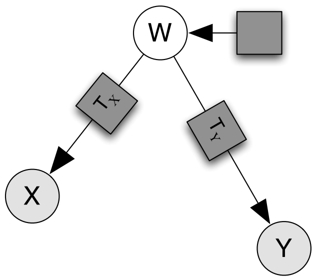

Figure 1. A simple example of transducer composition to build a multi-sequence model of two extant sequences. An ancestral sequence W evolves into two descendant sequences X and Y. A singlet transducer (the horizontal gray box) emit ancestral sequence and structure and two branch transducers (the gray boxes labeled ΔT_X and ΔT_Y) mutate it according to the specified multi-sequence model. Gray nodes correspond to observed data and white nodes unobserved data.

Stem creation. A stem is created at the root node W (corresponding to a bifurcation in the singlet transducer a_1) and survives in the sequence X at node 2 but is deleted in the sequence Y at node 3.

$$\begin{matrix} 1: \\ 2: \\ 3: \end{matrix} \quad \begin{pmatrix} L \\ L \\ \mathbf{L} \end{pmatrix} \rightarrow \begin{pmatrix} \mathbf{L} \\ W_L \\ W_L \end{pmatrix} \rightarrow \begin{pmatrix} B \\ B \\ B_p \end{pmatrix} \rightarrow \begin{pmatrix} L \\ L \\ \mathbf{L} \end{pmatrix} \begin{pmatrix} S \\ \mathbf{S} \\ \texttt{End} \end{pmatrix} \rightarrow \begin{pmatrix} L \\ L \\ \mathbf{L} \end{pmatrix} \begin{pmatrix} \mathbf{S} \\ W_S \\ \texttt{End} \end{pmatrix} \tag{1}$$

$$\rightarrow \begin{pmatrix} \mathbf{L} \\ W_L \\ W_L \end{pmatrix} \begin{pmatrix} \mathbf{S} \\ W_S \\ \texttt{End} \end{pmatrix} \tag{2}$$

Stem insertion. A stem sequence is inserted in sequence X at node 2.

$$\begin{matrix} 1: \\ 2: \\ 3: \end{matrix} \quad \begin{pmatrix} L \\ L \\ \mathbf{L} \end{pmatrix} \rightarrow \begin{pmatrix} L \\ \mathbf{L} \\ W_L \end{pmatrix} \rightarrow \begin{pmatrix} L \\ B_i \\ B \end{pmatrix} \rightarrow \begin{pmatrix} L \\ L \\ \mathbf{L} \end{pmatrix} \begin{pmatrix} \texttt{End} \\ S \\ \mathbf{S} \end{pmatrix} \rightarrow \begin{pmatrix} \mathbf{L} \\ W_L \\ W_L \end{pmatrix} \begin{pmatrix} \texttt{End} \\ \mathbf{S} \\ W_S \end{pmatrix} \tag{3}$$

Stem termination. All stem sequences are ended by (possibly empty) loop sequences.

$$\begin{matrix} 1: \\ 2: \\ 3: \end{matrix} \quad \begin{pmatrix} \mathbf{S} \\ W_S \\ W_S \end{pmatrix} \rightarrow \begin{pmatrix} B_e \\ B_e \\ B_e \end{pmatrix} \rightarrow \begin{pmatrix} \texttt{End} \\ \texttt{End} \\ \texttt{End} \end{pmatrix} \begin{pmatrix} L \\ L \\ \mathbf{L} \end{pmatrix} \rightarrow \begin{pmatrix} \texttt{End} \\ \texttt{End} \\ \texttt{End} \end{pmatrix} \begin{pmatrix} \texttt{End} \\ \texttt{End} \\ \texttt{End} \end{pmatrix} \tag{4}$$

The functions $\alpha(t)$, $\beta(t)$ and $\gamma(t)$ are parametrized by the insertion and deletion rates of the Structure Tree model. They are defined for loop sequences as

$$\kappa_1 = \lambda_1/\mu_1 \tag{5}$$

$$\alpha_1 = \exp(-\mu_1 t) \tag{6}$$

$$\beta_1 = \frac{\lambda_1 (1 - \exp((\lambda_1 - \mu_1)t))}{\mu_1 - \lambda_1 \exp((\lambda_1 - \mu_1)t)} \tag{7}$$

$$\gamma_1 = 1 - \frac{\mu_1 (1 - \exp((\lambda_1 - \mu_1)t))}{(1 - \exp(-\mu_1 t)) (\mu_1 - \lambda_1 \exp((\lambda_1 - \mu_1)t))} \tag{8}$$

and similarly for stem sequences [1].

References

1. Holmes I (2004) A probabilistic model for the evolution of RNA structure. BMC Bioinformatics 5.
